# Behaviour of the Pleistocene marsupial lion deduced from claw marks in a southwestern Australian cave

**DOI:** 10.1038/srep21372

**Published:** 2016-02-15

**Authors:** Samuel D. Arman, Gavin J. Prideaux

**Affiliations:** 1 School of Biological Sciences, Flinders University, Bedford Park, South Australia 5042, Australia

## Abstract

The marsupial lion, *Thylacoleo carnifex*, was the largest-ever marsupial carnivore, and is one of the most iconic extinct Australian vertebrates. With a highly-specialised dentition, powerful forelimbs and a robust build, its overall morphology is not approached by any other mammal. However, despite >150 years of attention, fundamental aspects of its biology remain unresolved. Here we analyse an assemblage of claw marks preserved on surfaces in a cave and deduce that they were generated by marsupial lions. The distribution and skewed size range of claw marks within the cave elucidate two key aspects of marsupial lion biology: they were excellent climbers and reared young in caves. Scrutiny of >10,000 co-located Pleistocene bones reveals few if any marsupial lion tooth marks, which dovetails with the morphology-based interpretation of the species as a flesh specialist.

When humans first set foot in Australia around 50,000 years ago they entered a unique landscape occupied by large reptiles, birds and mammals seen nowhere else. These included the anatomically-bizarre *Thylacoleo carnifex*. The ubiquity of this species and its evocative depiction in Aboriginal rock art^1^ suggest an important role in Australian ecosystems, but despite numerous skeletal studies, interpretations have remained controversial. The species was initially described by Richard Owen in 1859 as “one of the fellest and most destructive of predatory beasts”^2^, a view to which he was largely led by its greatly enlarged slicing premolar. It was soon after reinterpreted as a herbivore^3^, because it retained the herbivorous, diprotodontian template of enlarged first incisors, tiny canines, blade-like premolars, and large masseter and pterygoid muscles. Subsequently, the diet and behaviour of *T. carnifex* have been intensely debated. The species has been speculatively portrayed as a consumer of crocodile eggs^4^, a hyaena-like scavenger^5^, a melon specialist^6^, a leopard-like predator that dragged prey into trees^7^, a slow- to medium-paced runner incapable of climbing^8^, a terrestrial version of a cookie-cutter shark or raider of kangaroo pouches^9^, and a bear-like super-predator^10^. The doubts over how to interpret its bizarre combination of features are not due to a lack of fossil bones: *T. carnifex* is better represented in Pleistocene localities that any other large carnivore^11^,^12^, and more complete or partial skeletons are known from caves than for any other extinct Pleistocene species^8^,^12^,^13^,^14^. Although the current consensus is that *T. carnifex* was a carnivore, other lines of evidence are required to generate further insights into its behaviour and ecology.

Trace fossils, such as trackways or burrows, can provide insights into locomotory abilities and behaviours unobtainable via functional analyses of the skeleton alone. However, it is seldom possible to associate skeletal and trace fossils^15^. This underscores the significance of a claw-mark assemblage in the main chamber of Tight Entrance Cave (TEC), southwestern Australia (Fig. 1), where a now-blocked entrance in the ceiling provided access to the surface for species capable of navigating the steep, convoluted cave terrain. TEC also contains a diverse Pleistocene bone deposit^16^, which allows us to generate a shortlist of two extinct and five still-extant species of candidate claw markers based on known or potential climbing ability or cave utilisation. Past studies have noted cave surfaces scratched by Pleistocene cave bears^17^ and humans^18^, and fossil burrows scratched by their rodent^19^ and xenarthran^20^ makers. In particular the long deep marks of cave bears are thought to be associated with navigation in darker areas, or in locomotion through the complex 3D cave environment^21^. However, analyses have been largely qualitative and overlooked as a reliable information source beyond determining their maker. To analyse the TEC traces we pioneered a quantitative analysis involving comparisons of manual claw-mark dimensions with those made by living animals and simulated scratch sets of *T. carnifex*. We also sought evidence for tooth marks on bones, which we hypothesised may be evident if carnivores played a role in accumulating the bones.

## Results

### Claw-mark analysis

**Figure 1:**
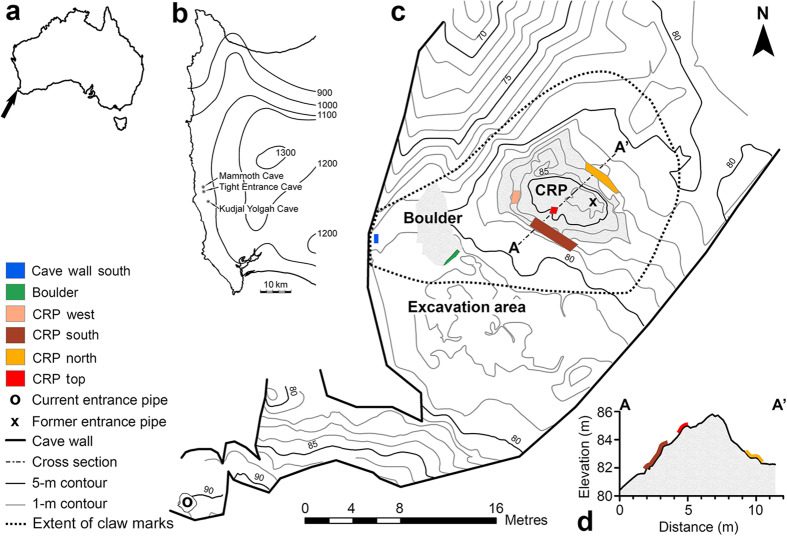
Location and map of Tight Entrance Cave. (**a**) Australia with arrow indicating position of locality. (**b**) Leeuwin–Naturaliste Region showing mean annual rainfall isohyets (mm) and caves containing palaeontological deposits preserving remains of *Thylacoleo carnifex*. (**c**) Plan view of main chamber showing focus areas for claw-mark documentation, excavation area and topography relative to sea level. (**d**) Cross section of central rock pile (CRP). The current entrance opened in 1976. The hypothesised former (Pleistocene) entrance in the main chamber ceiling is now blocked. Map generated in ArcMap10.1 using the CrossView extension; data collected on a Sokkia 3030R Total Station.

**Figure 2:**
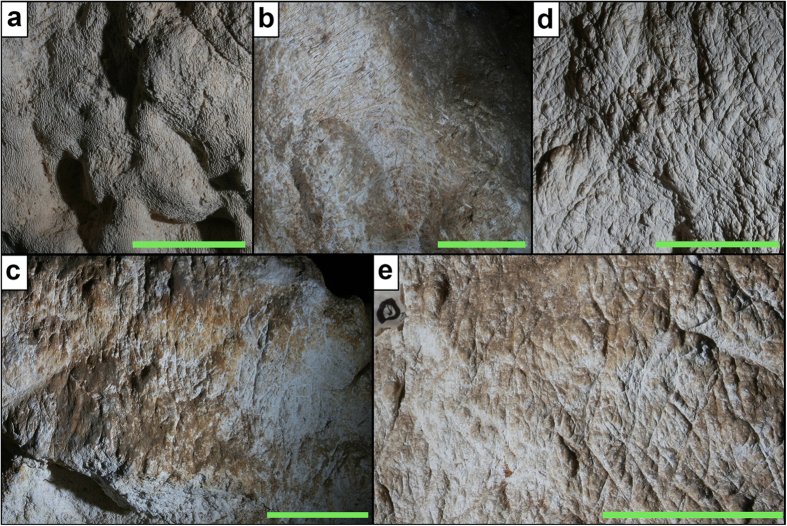
Examples of claw-marked areas in Tight Entrance Cave. (**a**) Cave wall south. (**b**) Central rock pile west. (**c**) Central rock pile south sub-region 2. (**d**) Boulder sub-region 2. (**e**) Boulder sub-region 8. Scale bars = 10 cm.

Thousands of claw marks, V-shaped in cross section, are patchily distributed through the TEC main chamber on a range of surfaces differing in hardness, including limestone boulders, mud encrustations and soft moonmilk (Fig. 1). The majority are located on the steep sides (40–90°) of the boulder and central rock pile, the scratched faces of which are up to 3.3 m in height (Fig. 1). However, the chaotic, superimposed nature of the assemblage means that only 103 were distinguishable within the six focus areas (Figs 1 and 2; Supplementary Information) as scratch sets, two or more claw marks made by a paw during the same substrate-contacting event. Sets vary markedly in orientation and length ranging from 5 to 150 mm, with a mean of 40 mm (Supplementary Table 10). Most sets (68%) were composed of two marks, with sets of three (22%) and four (10%) comprising the remainder. This resulted in recognition of 144 individual pairs, with most (55%) classified as parallel (Supplementary Table 10). In the event that these marks could be confidently attributed to *T. carnifex*, we addressed the degree to which this lateral digital movement might have been facilitated by the enlarged semi-opposable digit I alone by focusing on scratch sets composed of >1 pair of marks, and subsequently eliminating the largest IDS (i.e., that potentially between digits I and II). This revealed that 16 of 43 pairs converged or diverged >20% more than the IDS mean for *T. carnifex* generated from the mock actualistic trial (Supplementary Tables 9 and 11).

**Figure 3:**
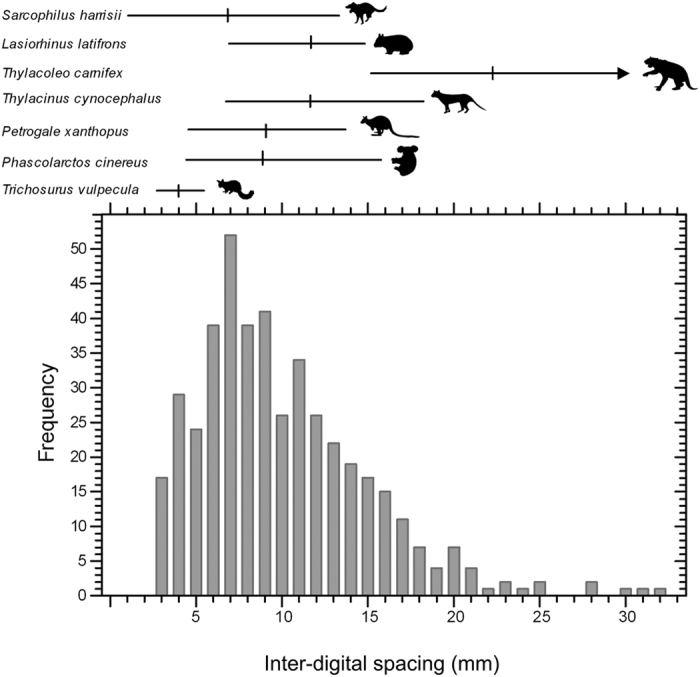
Histogram of inter-digital spacing for the Tight Entrance Cave scratch sets. Lines above represent the ranges exhibited by the taxa considered in the comparative study, with vertical lines representing means. Arrow on *Thylacoleo carnifex* indicates that this extends beyond the range of the histogram. Silhouettes not to scale. Sourced from Phylopic.org, all under public domain except: *Sarcophilus* (http://phylopic.org/image/58cc56c2-5a36-4031-be9f-28c86f77963c/) by Sarah Werning; *Thylacinus* (http://phylopic.org/image/bacd7beb-7b6f-4466-a31a-715509b9532f/) by Michael Ströck; and *Petrogale* (http://phylopic.org/image/eccfc4b3-faee-4384-b35b-64c788f30846/) by T. Michael Keesey, all unchanged under Creative Commons Attribution-ShareAlike 3.0 Unported licence. The license terms can be found at: https://creativecommons.org/licenses/by-sa/3.0/.

Actualistic and opportunistic studies of modern species allowed each to largely be differentiated on the basis of claw-mark attributes in combination with behavioural information (Supplementary Tables 1 and 3). Wombat claw marks are distinguished from those of all other species by their U-shaped cross-section (Supplementary Fig. 4). Koala (*Phascolarctos cinereus*) claw marks fall into two groups: longer descent and shorter ascent marks (Supplementary Fig. 5). Those left on trees by the Common Brushtail Possum (*Trichosurus vulpecula*) are consistently very small (inter-digital spacing [IDS] range 2.7–5.2 mm) and only lightly impressed into even relatively soft bark (Supplementary Fig. 5). The claw-mark morphology and range of IDS values exhibited by the Yellow-Footed Rock-Wallaby (*Petrogale xanthopus*), Tasmanian Devil (*Sarcophilus harrisii*) and Thylacine (*Thylacinus cynocephalus*) samples overlap and fall within the smaller half of the TEC range (Fig. 3). Marks made by the replica adult manus of *T. carnifex* have IDS values that are distinctly larger than for all other species.

### Tooth-mark analysis

**Figure 4:**
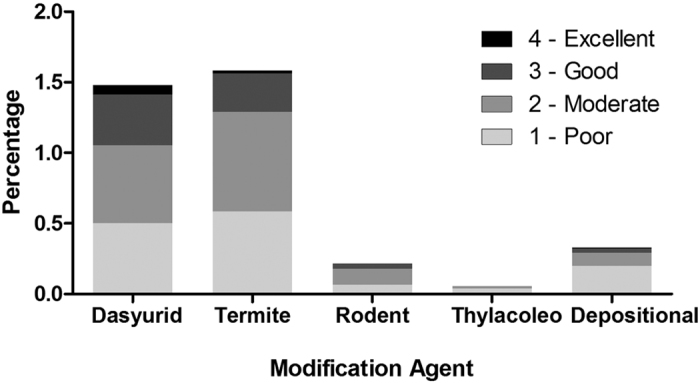
Histogram of bone modification types from Tight Entrance Cave indicating confidence with which marks were attributed to biological agents (Reliability index). Percentage refers to that of all bones inspected for taphonomic damage (10,621).

Only 2.3% of the 10,621 TEC bones examined show any evidence of post-mortem surface marks potentially made by a biological agent (Fig. 3). Less than half of these bear features that may represent tooth marks of *T. carnifex* (n = 2) or *S. harrisii* (n = 103). Considering only those marks with a reliability index of good or excellent (Fig. 4) eliminates all except 45 bones (0.43%) with marks attributed to devils. The 45 devil-marked bones are too large or complete to have been derived from scats (Supplementary Table 15), and indeed no fragments consistent with a scat signature have been recognised among the entire TEC sample.

## Discussion

The largest TEC ichnofossil scratch marks can only have been made by *T. carnifex.* Smaller marks may be attributable to a number of agents, but juvenile *T. carnifex* seems the most likely given the known biology of species present and comparisons with trogloxene behaviour elsewhere. The smaller TEC claw marks bear a cursory resemblance to the longer descent marks made by koalas, but the sheer number of marks is more consistent with one or more species of trogloxene rather than individuals of an obligate arboreal folivore that may have unwittingly entered the cave. While common brushtail possums are opportunistic trogloxenes, the light marks that they habitually leave on bark and on cave surfaces^22^ are not consistent with the deep scratches observed in TEC. Use of caves for shelter is known for rock-wallabies, thylacines and devils^23^,^24^, but while rock-wallabies are highly adept in steep terrain, they primarily use their hind limbs to move ricochetally. No TEC marks match the broad macropodid pedal claw morphology. Rock-wallabies are capable of transferring weight to their forepaws to pivot on ledges during ascent of rocky slopes, but most of the weight is borne on the pads. The large volume and broad distribution of TEC claw marks are more consistent with primarily quadrupedal animals for which manual claw use is critical. Only some aspects of the biology of the thylacine were documented prior to its extinction in 1936, but there is no record of climbing and its limbs lack any features indicative of a scansorial ability^25^. By contrast, devils, especially juveniles, are capable climbers^23^.

The modern devil IDS range coupled with the observation that Pleistocene *S. harrisii*, like several of its contemporaries, was smaller in the west^26^, suggest that only marsupial lions could have generated scratch sets with IDS values >14 mm. Devils could feasibly have contributed to the smaller end of the spectrum. However, IDS ranges for the modern species are normally distributed whereas the TEC range, although unimodal, is strongly positively skewed (Fig. 3). This could imply that the claw-mark assemblage was produced by both marsupial lions and devils, or that marsupial lions alone produced the assemblage, with most claw marks made by juveniles. The latter would be consistent with use of the cave as a maternal den to protect and raise young as observed in hyaenas and large dasyurid marsupials, e.g., devils and spotted-tailed quolls (*Dasyurus maculatus*)^23^,^27^,^28^. Similar suggestions have been made on ichnofossil scratch marks in a Romanian cave, where a range of scratches of width 5–12 cm (across 4 digits) was interpreted as indicating cave bears of different ages^17^.

Further insight is provided by the taphonomic analysis, which reveals minimal evidence of carnivore tooth damage. Devils either accumulated a small fraction of bones themselves or, more likely, occasionally modified the remains of animals that entered the cave via a different agency. Feeding studies of devils show that they ingest most bone at or near a kill or scavenge site; an abundance of fragments derived from scats is the hallmark of their cave dens^29^,^30^. The rarity of chewed bones and absence of scat-derived fragments reinforces the view that devils were a negligible accumulator^31^. The lack of *T. carnifex* tooth marks might also reflect their minimal role in collecting bones: the relatively complete preservation of most bones, broad size range of taxa and high species richness has been used to interpret the deposit as a pitfall accumulation, with animals falling through now-blocked solution pipes along with the sediments that entombed them^16^. This situation parallels that seen in a number of European caves where cave bears, hyaenas and lions often occupied caves as fossil remains accumulated^17^,^32^. The differential evidence of den use also parallels that in Australia: scavenging devils and hyaenas leave traces on chewed bones; *Thylacoleo* and cave bears leave scratch marks on cave surfaces; thylacines and lions are principally inferred from faunal remains^17^. The lack of tooth marks on TEC bones attributable to *T. carnifex*, however, may also reflect the morphology-based interpretation of this species as a flesh specialist^7^,^10^. Tooth marks on bones attributable to marsupial lions are only sporadically encountered and apparently incidental to dragging about carcasses or stripping them of meat and viscera^7^,^33^. This is exemplified today by lions, where incidental marking of bone during flesh stripping is common at kill sites, but rarely substantial enough to be seen in the fossil record^34^.

The most parsimonious interpretation of the TEC evidence is that marsupial lions were primarily responsible for the claw-mark assemblage. Devils clearly used the cave at times during the depositional interval of 140–30 kyr ago, but it is improbable that the two species cohabited the chamber given the intense interspecific antagonism displayed by carnivores, especially in cave settings^17^. With an estimated adult body mass of 80–100 kg, *T. carnifex* was 10–15 times heavier than *S. harrisii* (6–8 kg), which, even in a juvenile-biased mob, would likely have been at a major competitive advantage within a prime den environment. In addition, despite the number of documented devil dens, and known devil climbing behaviour, no den scratch marked by devils has yet been documented. The palimpsest-like nature of the TEC trace-fossil assemblage does not allow us to tightly constrain the span over which claw marks accumulated, but it may be as great as 90 kyr, given that the latest record of *T. carnifex* within the TEC bone deposit is in unit E, dated to 51 ± 2 kyr^16^. In addition, archaeological marks on moonmilk have been shown to degrade over time^35^, so the fidelity of marks on this material suggests no great age for the marks.

The distribution of claw marks indicates a significant bias toward juveniles. One possibility is that, at any one time, the chamber was occupied by a lone mother looking after a single litter, as in *D. maculatus*, where a female may raise up to six young in a cave den^28^. Alternatively, the communal arrangement exemplified by brown or spotted hyaenas involving multiple reproductive females^36^ may be a better model. The latter draws circumstantial support from the now-largely-destroyed Komatsu Cave in southeastern Australia, which preserved a series of articulated partial skeletons, including a directly associated putative adult female and juvenile individuals, and a claw-mark assemblage^14^,^37^,^38^.

Marsupial lions, like all marsupials, would have given birth to extremely underdeveloped young that could not be left alone until becoming at least partially weaned. Adult female body mass and time to weaning is correlated in marsupials^39^. For thylacines (estimated adult body mass 15–35 kg), young spent three months in the pouch followed by a further month of semi-independence^24^. Marsupial lion females were at least three times the adult body mass of thylacines, which suggests that they may have borne young in the pouch for a minimum of four months. Carrying older pouch young while hunting probably constrained predatory efficiency or prey size range. Even without invoking the difficulties involved in carrying pouch young, mere accompaniment by cubs has been implicated in 16% of failed chases by cheetahs (*Acinonyx jubatus*)^40^. As in numerous extant carnivores, adult marsupial lions likely left semi-independent young to shelter in the cave while they went off to hunt before returning to bring food and to rest. This is precisely what is seen in living *S. harrisii*: once weaned, juveniles are left in a lair while the mother hunts^41^. The TEC claw-mark size distribution shows that, as individuals grew, they spent less time in the cave, presumably venturing out increasingly to learn from adults and contribute to hunting.

Many claw marks within TEC are located on steep surfaces, despite more gradual inclines being available on other sides of the central rock pile and boulder (Fig. 1). This suggests regular, confident, purposeful climbing with a high degree of agility. Climbing has similarly been invoked for a small number of *Ursus spelaeus* scratch marks high on a Romanian cave wall^17^. This distribution reinforces the argument, based on skeletal morphology, that *T. carnifex* could climb trees^7^,^37^. This is in spite of its large size and “bear-like” build^10^, which have been used to argue against its arboreal adeptness^9^. Looking at the ecologically comparable carnivorans, small size does correlate with climbing ability, but 7 of 61 species studied weighed 80kg or more and were considered capable climbers^42^. The density of marks on the central rock pile also points toward the most feasible Pleistocene entrance. Above the eastern end of its apex is a now-blocked, 0.7-m-wide solution pipe, the lowermost 1 m of which is clear of sediment and slopes at approximately 45° to the ground surface 6 m above. Subsidence, possibly prior to the deposition of units H and J (40–30 ka), lowered the central rock pile^31^ such that its apex is now 3 m below the solution pipe, prohibiting animals from exiting the cave. Prior to this time the cave would have acted as a single large chamber, accessible to the surface by marsupial lions, while acting as a faunal trap for species unable to climb out, more similar to a European open hyaena cave than the deep hibernation chamber favoured by cave bears^32^.

Marked variance in scratch-set orientation on steep surfaces reflects substantial limb mobility consistent with the high degree of abduction inferred from functional morphological analysis^7^. Similarly, the convergence and divergence exhibited by many scratch sets points to greater lateral movement of digits II–IV than has been suggested from analysis of metacarpal–phalangeal articular morphology^43^. These attributes would have combined to aid in gaining traction during climbing. This again is consistent with the condition in carnivorans, where more curved claws, and higher metacarpal/phalanx ratio are associated with climbing taxa, assisting in providing a broad grip^42^. Extensive claw marking on horizontal surfaces atop the TEC central rock pile indicates that claw engagement with the substrate was able to be maintained when not climbing. By contrast, fossil manus prints of *T. carnifex* from a lacustrine environment in southeastern Australia preserve four distinct impressions made by digits II–V^15^. The absence of claw marks there shows that claws could also be raised when moving on a relatively flat surface. Thus, claw engagement when moving across flat surfaces was likely context-dependent, and perhaps more likely to occur in a more unstable or dark environment. The paucity of sets composed of >3 claw marks, and in particular those where one of the outside IDS mean values is substantially greater than the others, reflects infrequent use of digit I during climbing within the cave. This observation, and the fact that the southeastern cave wall manus prints lack digit I impressions (see Supplementary Fig. 7), supports the suggestion, based on manual morphology, that digit I could be raised during terrestrial locomotion^43^, likely coming into play more when grappling with prey or tree climbing. This feature also highlights a major difference between the scratch marks made by cave bears and marsupial lions where the former almost exclusively leave indications of four digits^17^, whereas the latter rarely left sets of >3 marks. This may further reflect the more dynamic manus arrangement in *T. carnifex,* compared to the more static cave bear paw.

The TEC trace-fossil evidence shows that *T. carnifex* was highly proficient at negotiating a dark, complex environment. Most claw marks are on highly heterogeneous or steep surfaces, providing clear behavioural evidence to support morphological studies inferring that *T. carnifex* could climb trees^37^,^43^. That claw marks of younger individuals dominate the assemblage indicates that TEC was utilised for raising and protecting young over an extended duration. This supports an idea advanced half a century ago^29^, but hitherto unverified, that marsupial lions used caves as dens where available, which likely explains the noted overabundance of their remains in cave skeletal assemblages^11^,^14^.

Dens are used by hyaenas to assist raising of young over extended periods of time. For hyaenas this is facilitated by their large groups, which help to defend resources from other groups^44^. This reflects that hyaena groups are strongly related, so that the shared costs of fights through fatalities and injuries are outweighed by the benefit to the group as a whole^44^. In contrast, solitary or small families of bears use caves primarily during the winter for hibernation, but also as cool places in summer, while having additional security benefits^45^. The element of security is one factor uniting these two ecologically distinct trogloxenes, as well as fitting palaeontological reconstructions from Europe^17^. Caves provide a safe, temperature controlled environment, and would hence be a sought after resource. With a high proportion of juvenile marsupial lions in TEC, communal living as is seen today in hyaenas seems the most plausible arrangement to defend this resource from both other denning marsupials (thylacines and devils) as well as other conspecific groups.

The absence of tooth marks on TEC bones attributable to *T. carnifex* reinforces the idea the deposit represents a pitfall accumulation, and is consistent with the dentition-based interpretation of *T. carnifex* as a flesh specialist. The high bite strength and advanced meat-slicing capabilities of *T. carnifex* represent the most extreme manifestation of the tendency for large, felid-like taxa to evolve where felids are absent^46^. By comparison, the second-largest Australian mammalian carnivore (thylacine) hunted individually or in pairs and focused on smaller prey^47^. Given that marsupial lions were apparently adapted to apprehending and consuming large prey^10^ and potentially social, it is feasible that, as in all extant group-living mammalian predators^35^, they were cooperative hunters. As body mass, group living and group hunting are seen as co-adaptations for procuring large prey^48^, it is plausible that marsupial lions were pack hunters. Such a strategy would have allowed them to prey upon the largest marsupial, the rhinoceros-sized *Diprotodon optatum*, bones of which have been found with incidental marsupial lion tooth marks^33^.

## Methods

### Comparative analysis

Species were shortlisted based on their presence in the TEC bone assemblage^16^, paw morphology, and known behavioural and locomotory attributes (Supplementary Table 1). Actualistic studies, where markings made by captive animals were measured, were undertaken on captive individuals of *Sarcophilus harrisii* and *Lasiorhinus latifrons*, which was used as an extant analogue for *Vombatus hacketti*. Bark scratched during the arboreal activities of *Trichosurus vulpecula* and *Phascolarctos cinereus* in the Adelaide foothills was opportunistically collected and measured. For each scratch set, digit width (width of each claw mark) and inter-digital spacing (distance between adjacent marks) were measured using digital calipers or photogrammetry in ArcMap 10. For *Thylacinus cynocephalus, Petrogale lateralis* and *Thylacoleo carnifex* (replica), an articulated manus was drawn across clay (Supplementary Fig. 6) and the resultant scratches measured. In actualistic and opportunistic studies, the length of the scratch set was also measured. Actualistic experiments were carried out at Zoos SA in accordance with their behavioural enrichment program. All experimental protocols were approved by W. Foster, Manager of Conservation Programs.

### Trace-fossil documentation

Scratched surfaces were surveyed using a Total Station as part of a cave survey. Photos of surfaces were georectified to the survey data in ArcMap10 and visually inspected for marks distinguishable into clear sets (Supplementary Information). Digit width was not measured because of the high irregularity of claw mark edges on limestone surfaces. Individual scratch marks were digitally traced, and measurements extracted using the ‘calculate geometry’ tool for spacing and length. Set orientation was extracted by tracing out polygons covering each scratch set, and measured using the ‘Calculate Polygon Main Angle’ tool. Marks were ascribed to species on the basis of scratch-set attributes and consideration of the known biology of the candidate species. Because data were non-normally distributed, a Kruskal–Wallis test was used to compare scratch sets across six regions. Transformations were not considered because variance from normality was not uniform across regions. Mann–Whitney pairwise comparisons were used to compare individual regions. Analyses were conducted in PASW Statistics 18.0 and PAST 2.07 < http://folk.uio.no/ohammer/past/>.

### Taphonomic analysis

TEC fossils are stored in the Department of Earth and Planetary Sciences, Western Australian Museum, Perth. A total of 1,344 registered and 9,277 unregistered specimens were inspected for marks evidently made by biological agents. Only specimens of species within the potential prey-size range of *Sarcophilus harrisii* and *Thylacoleo carnifex* were considered (>3 kg adult body mass). We ignored marks evidently caused by trampling and excavation trauma. Diagnosis of bone modifications were guided by published descriptions^5^,^30^,^33^,^49^ and divided into five biological modification agents: *S. harrisii*, *T. carnifex*, murid rodent and termite (Supplementary Table 12). Depositional markings often mistaken for animal bone modifications, e.g., root etching, were also identified. A reliability index was developed to denote the confidence with which markings could be attributed to perpetrator (Supplementary Table 13).

## Additional Information

**How to cite this article**: Arman, S. D. and Prideaux, G. J. Behaviour of the Pleistocene marsupial lion deduced from claw marks in a southwestern Australian cave. *Sci. Rep.* **6**, 21372; doi: 10.1038/srep21372 (2016).

## Supplementary Material

Supplementary Information
