# Supplementary material for: Behaviour of the Pleistocene marsupial lion deduced from claw marks in a southwestern Australian cave
Source: Sci Rep. 2016 Feb 15;6:21372. doi: 10.1038/srep21372 (PMC4753435; doi:10.1038/srep21372)
Supplement: Supplementary Information [file srep21372-s1.doc]

**SUPPLEMENTARY INFORMATION**

**Behaviour of the Pleistocene marsupial lion deduced from claw marks in a southwestern Australian cave**

Samuel D. Arman^1^ & Gavin J. Prideaux^1*^

*1 School of Biological Sciences, Flinders University, Bedford Park, South Australia 5042, Australia*

** To whom correspondence should be addressed. E-mail: gavin.prideaux@flinders.edu.au*

**Supplementary Figure 1** Skeletal reconstruction of the marsupial lion, *Thylacoleo carnifex*.

**Supplementary Figure 2** *Sarcophilus harrisii* actualistic trial setups.

**Supplementary Figure 3** Claw-marked boards resulting from *Sarcophilus harrisii* actualistic trial.

**Supplementary Figure 4** Claw marks recorded in the *Lasiorhinus latifrons* actualistic trial.

**Supplementary Figure 5** Opportunistically-collected bark claw marked by *Trichosurus vulpecula* and *Phascolarctos cinereus*.

**Supplementary Figure 6** Mock claw marks of *Thylacoleo carnifex* in modelling clay.

**Supplementary Figure 7** Claw-marked surface, Cave Wall South, Tight Entrance Cave.

**Supplementary Figure 8** Boulder sub-regions within which scratch sets were analysed.

**Supplementary Figure 9–16** Overview of claw-marked surfaces on Boulder, Tight Entrance Cave.

**Supplementary Figure 17–29** Overview of claw-marked surfaces on Central Rock Pile, Tight Entrance Cave.

**Supplementary Figure 30** Examples of bones from Tight Entrance Cave modified by biological agents.

**Supplementary Table 1** Potential claw-marking species shortlisted for comparative analysis.

**Supplementary Table 2** Categorisation formulae used to indicate morphology of claw marks.

**Supplementary Table 3** Summary claw-mark data from comparative analysis.

**Supplementary Table 4** Summary information on the *Sarcophilus harrisii* actualistic trial setup.

**Supplementary Table 5** Claw-mark data from *Sarcophilus harrisii* actualistic scratching trial.

**Supplementary Table 6** Claw-mark data from *Lasiorhinus latifrons* actualistic scratching trial.

**Supplementary Table 7** Claw-mark data for *Trichosurus vulpecula*.

**Supplementary Table 8** Claw-mark data for *Phascolarctos cinereus*.

**Supplementary Table 9** Claw-mark measurements from the *Thylacoleo carnifex* mock actualistic trial.

**Supplementary Table 10** Summary of Tight Entrance Cave claw-mark characteristics.

**Supplementary Table 11** Mann–Whitney pairwise comparisons across the Tight Entrance Cave claw-marked regions.

**Supplementary Table 12** Criteria used for analysis of Tight Entrance Cave bone modification by biological agents.

**Supplementary Table 13** Reliability index indicating degree of confidence with which markings on Tight Entrance Cave bones have been attributed to biological agent.

**Supplementary Table 14** Number and percentages of specimens in the Tight Entrance Cave bone assemblage noted to have been modified by biological agents grouped according to their Reliability index.

**Supplementary Table 15** Biologically-modified specimens (with Reliability Index 2–4) organised by unit.

**Supplementary Table 16** List of specimens bearing tooth marks of *Sarcophilus harrisii* with Reliability index 3–4.

**Supplementary Table 17** List of all specimens bearing potential tooth marks of Thylacoleo carnifex

**Supplementary Figure 1 | Skeletal reconstruction of the marsupial lion, *Thylacoleo carnifex***. (**A**) Anterior view. (**B**) Lateral view. Reconstruction by C. Burke and R. Wells. Photo credit: G. Prideaux.

**A**
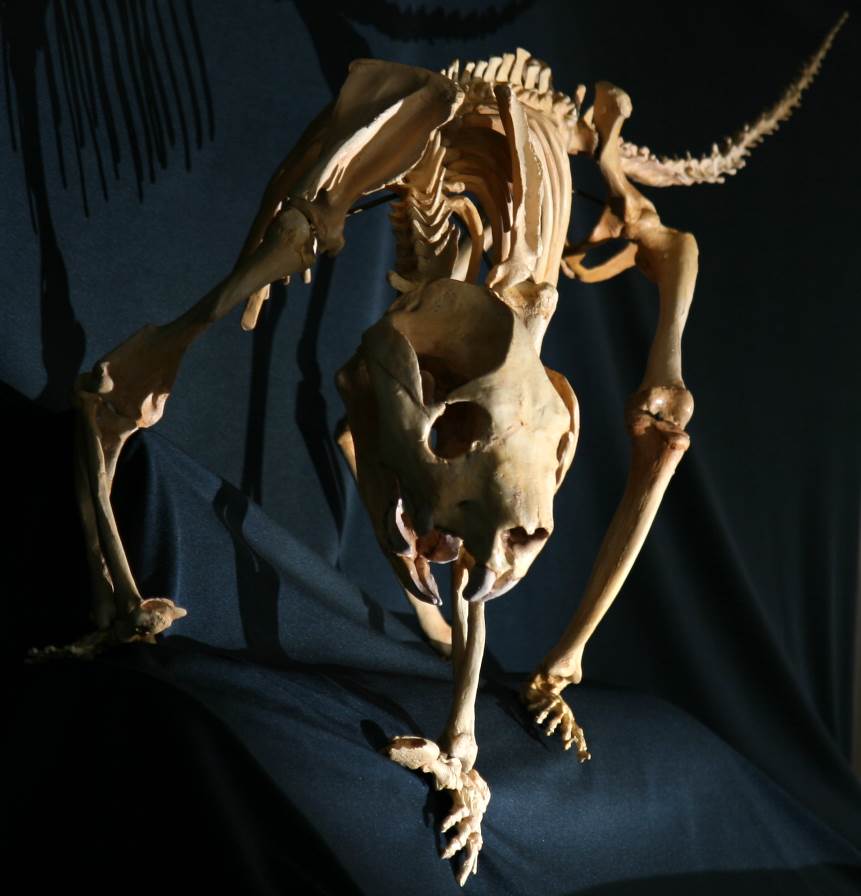


**B**
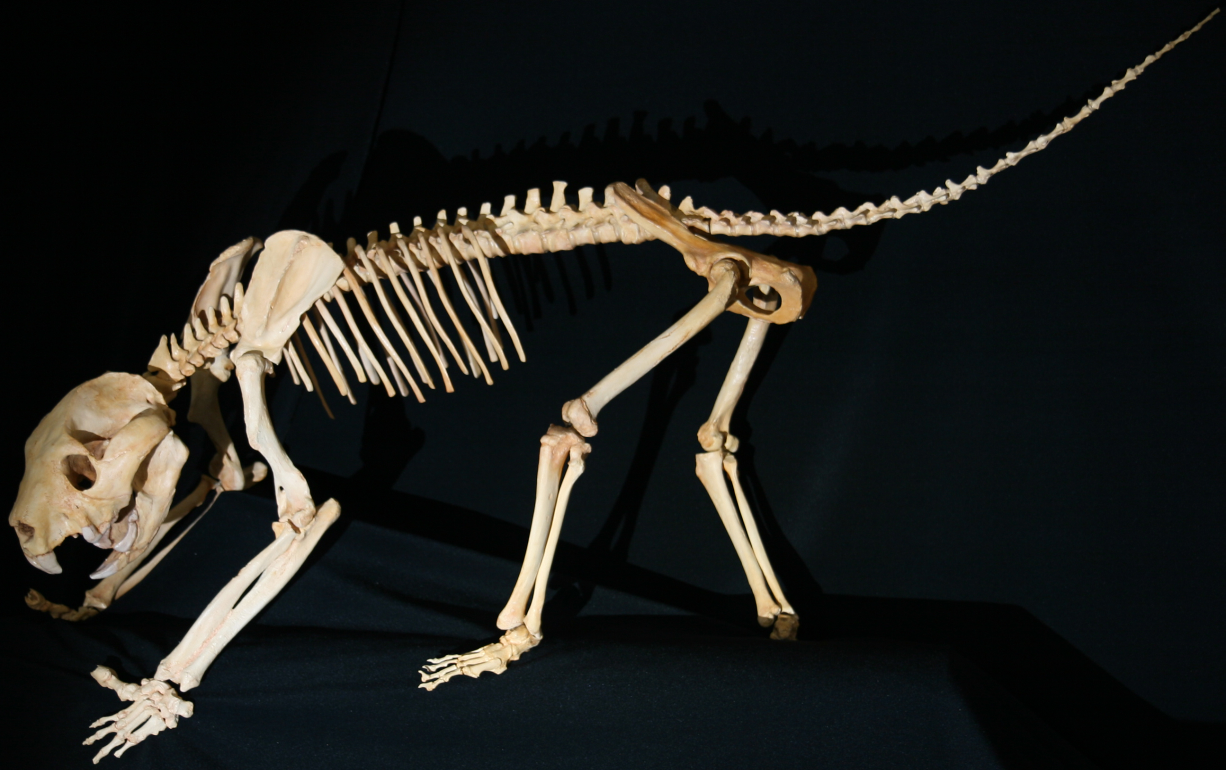


**Supplementary Table 1 | Potential claw-marking species shortlisted for comparative analysis. † = extinct. See Prideaux et al. (2010) for the full species list from the Tight Entrance Cave bone deposit.**

| **Species** | **Behavioural Basis for Inclusion** | **References** |
| --- | --- | --- |
| Tasmanian Devil, *Sarcophilus harrisii* | Climbing ability, cave habitation | Owen & Pemberton (2005) |
| Southwest Wombat, *Vombatus hacketti* † | Fossorial habit | Murray (1998) |
| Koala, *Phascolarctos cinereus* | Excellent climbing ability | Van Dyck & Strahan (2008) |
| Common Brushtail Possum, *Trichosurus vulpecula* | Excellent climbing ability, cave habitation | Bednarik (1991) |
| Black-flanked Rock-wallaby, *Petrogale lateralis* | High agility, cave habitation | Van Dyck & Strahan (2008) |
| Thylacine, *Thylacinus cynocephalus* † | Cave habitation | Paddle (2000) |
| Marsupial Lion, *Thylacoleo carnifex* † | Postulated climbing ability, postulated cave habitation | Lundelius (1966); Wells *et al.* (1982) |

**Supplementary Table 2 | Categorisation formulae used to indicate morphology of scratches.** U=uppermost mark; C= centre mark; L= lowermost mark, ≈ implies ≤2 mm difference.


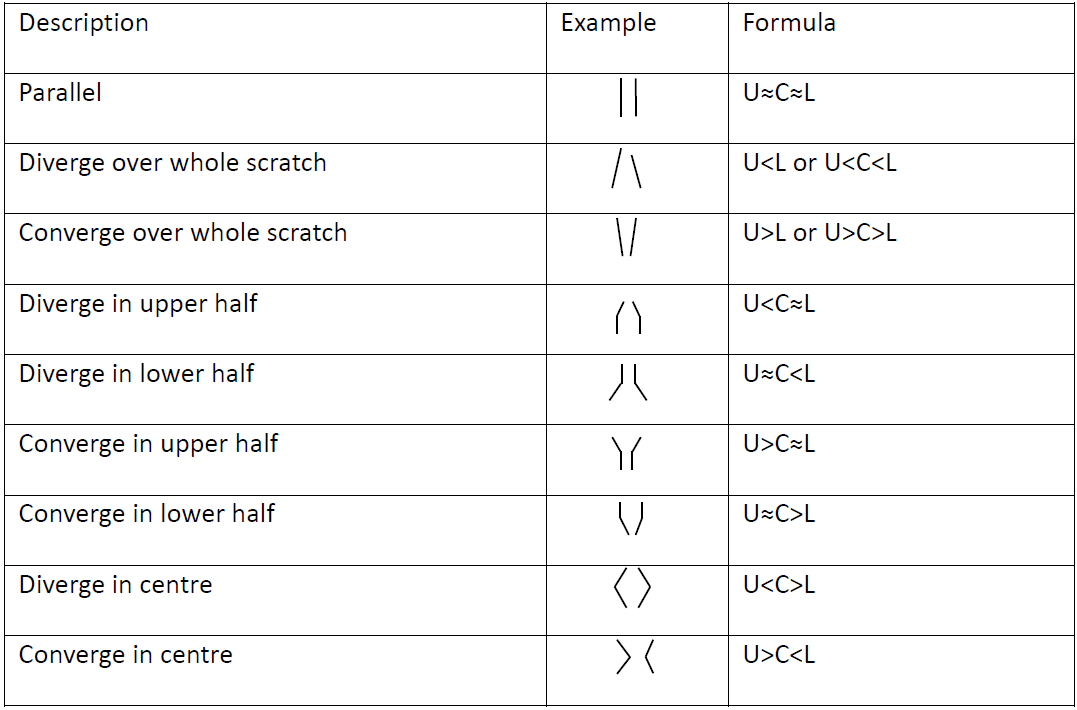


**Comparative Analyses**

***Methods***

*Sarcophilus harrisii—*Seven highly active individuals of an Adelaide (Monarto) Zoo population of breeding individuals were identified by keepers as good candidates for the actualistic study. Sheets of balsa wood were installed in their enclosures on small trees, logs and artificial ramps where the devils had been noted to climb (Supplementary Fig. 2). Food was placed above some of the boards and blood was also smeared upon and surrounding them to encourage investigation. Once the boards were retrieved, scratch sets were identified and measurements taken using digital calipers. To consider how inter-digital spacing (IDS) reflected the actual morphology of the marks, a simple categorisation procedure was utilised to define scratch morphologies (Supplementary Table 2). The average angle of sets relative to vertical was also documented using a protractor.

**Supplementary Figure 2.** Monarto Zoo *Sarcophilus harrisii* actualistic trial setups.(**A**) Station 1. (**B**) Station 2. (**C**) Station 7. (**D**) Station 6. (**E**) Station 5. (**F**) Station 4.


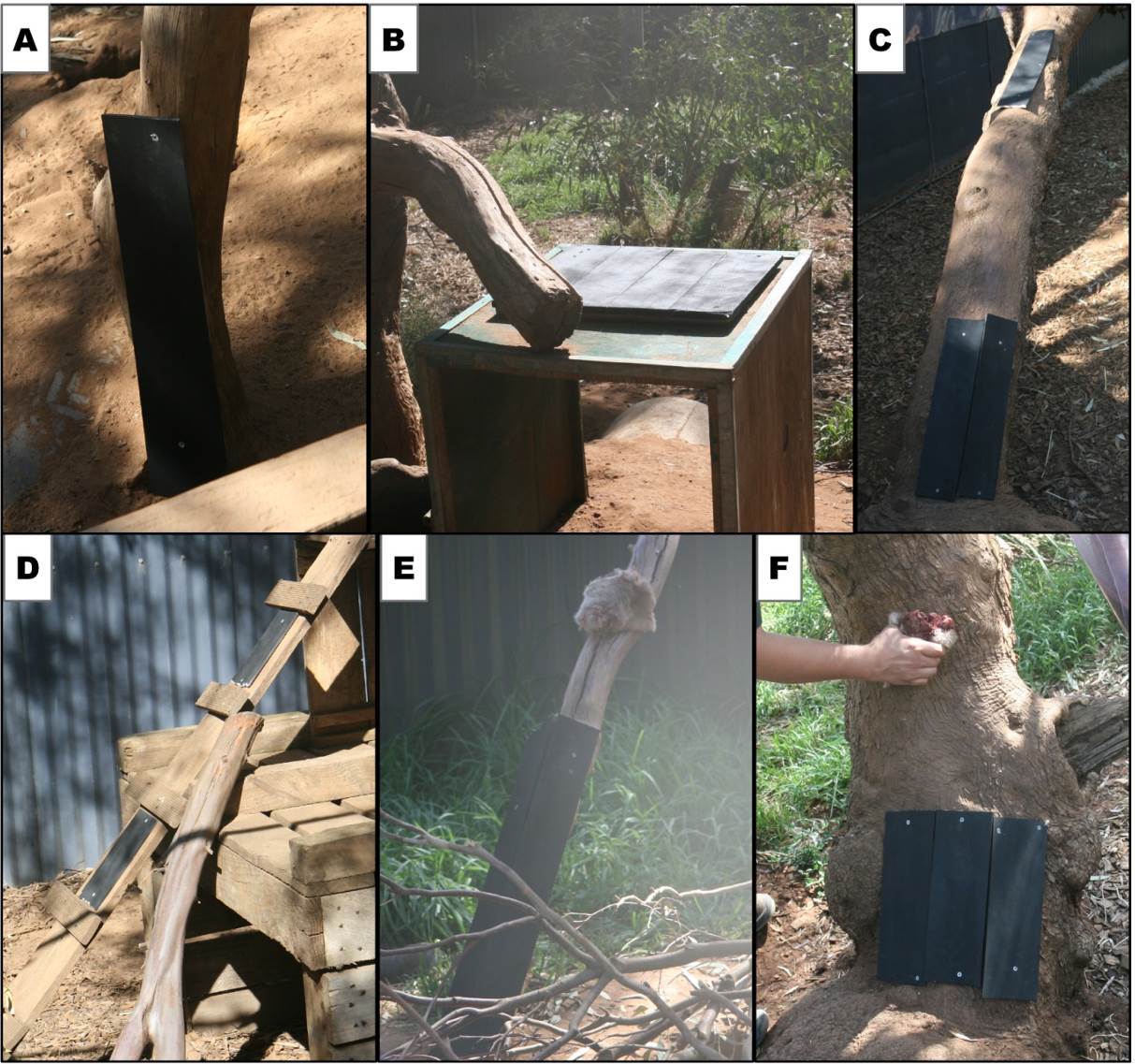


*Lasiorhinus latifrons—*Two Southern Hairy-nosed Wombats at the Adelaide Zoo were used as a proxy for extinct *Vombatus hacketti*. Instead of introducing material into the enclosure, which keepers feared was more likely to be chewed and eaten rather than scratched by the animals, it was decided that recording scratches made on the soil of the enclosure would be an easier and safer alternative. Areas where keepers had noted the animals preferred to dig were identified, sprayed with water and flattened the evening before recording. The following morning as the wombats were removed from the enclosure for feeding, marks were photographed and the area surveyed with a Total Station. Photographs were georectified to survey data and measurements taken in ArcMap 10. As the marks were made on a horizontal surface, no orientation data were recorded.

*Phascolarctos cinereus* and *Trichosurus vulpecula.*—Claw-marked bark was opportunistically collected from the Belair region of the Adelaide hills. Of the species present in this region (van Dyck & Strahan 2008), only the arboreal *Trichosurus vulpecula* and *Phascolarctos cinereus* are capable of climbing trees to produce the markings*.* Determining which species created the marks was undertaken by visual examination in comparison with published markings (Triggs 2004). Measurements were taken using digital calipers and orientation using a protractor, with the orientation of the bark used to approximate vertical.

*Thylacinus cynocephalus* and *Petrogale xanthopus—*Despite *Thylacinus* being only recently extinct, attaining a manus for measurement presented some difficulty due to the fragile nature of the South Australian Museum specimens. To overcome this, a specimen was scanned using X-ray micro-computer tomography (micro-CT) and printed in a three-dimensional model. A recently deceased adult female *Petrogale xanthopus* specimen donated to the Flinders University Palaeontology Laboratory by Cleland Wildlife Park in April 2010 for inclusion in the comparative collection was also used. Both were measured with digital calipers.

*Thylacoleo carnifex—*An articulated cast *Thylacoleo* manus was run across soft modeling clay, comparable to the methodology undertaken by Gobetz (2006). Two different configurations were used to replicate the large extension available to digit I (Wells & Nichol 1977)*.* Measurements were taken using digital calipers.

**Supplementary Table 3 | Summary claw-mark data from comparative analysis.** Characteristic features are attributes unique to each taxon. Dimensions for *Phascolarctos cinereus* refer to the shorter, more common claw-mark morphology (see Methods). All measurements in mm.

| **Species** | **Method** | **N** | **Digit Width** | | **Inter-Digital Spacing** | | **Length** | | **Characteristic Features** |
| --- | --- | --- | --- | --- | --- | --- | --- | --- | --- |
|  |  |  | RANGE | MEAN | RANGE | MEAN | RANGE | MEAN |  |
| *Sarcophilus harrisii* | Actualistic | 18 | 0.5-1.6 | 1.0 | 1.0-13.3 | 6.9 | 5-100 | 30.6 | None |
| *Lasiorhinus latifrons* | Actualistic | 6 | 7-11 | 8.7 | 7-15 | 11.5 | 100-190 | 143.3 | U-shaped markings in cross section |
| *Phascolarctos cinereus* | Opportunistic | 10 | 0.5-1.0 | 0.8 | 4.2-15.8 | 8.6 | 10-45 | 25.0 | Two scratch morphologies |
| *Trichosurus vulpecula* | Opportunistic | 2 | 0.4-0.6 | 0.5 | 2.7-5.2 | 4.0 | 10-15 | 12.3 | Light scratches |
| *Thylacoleo carnifex* | Mock Actualistic | 1 | 1.5-3.0 | 2.4 | 15-60 | 23.3 | - | - | Large IDS range due to high flexion of digit I |
| *Thylacinus cynocephalus* | Measured Cast | 1 | 4.8-6.6 | 5.7 | 6.8-17.9 | 11.4 | - | - | - |
| *Petrogale xanthopus* | Measured specimen | 1 | 2.2-3.3 | 2.7 | 4.5-13.8 | 8.8 | - | - | - |

***Results***

*Sarcophilus harrisii*—Multiple scratch sets were made by individuals during the trial (Supplementary Fig. 2) and measured (Supplementary Table 4). To understand the variation present in this sample, the IDS set length and orientation histograms (not figured) revealed normal distributions with a slight positive skew. This was supported by the Shapiro–Wilk test, which could not reject the null hypothesis of a normal distribution (*p* = 0.210). The set length histogram is more difficult to interpret, largely due to the binning of values into 5-mm intervals which created an apparent bi-modal distribution. This was supported by the Shapiro–Wilk test which refuted a normal distribution (*p* < 0.000). The orientation histogram indicated a slight positive skew, however a Shapiro–Wilk test could not refute the null hypothesis of a normal distribution (*p* = 0.265). The calculations undertaken classified two-thirds (22 of 33) of the markings as parallel, with no clear trends evident in the remaining data.

**Supplementary Table 4 | Summary information on the *Sarcophilus harrisii*** actualistic trial setup.

| **Station** | **Size of Boards (cm)** | **Enclosure** | **Age** | **Sex** | **Location** | **Notes** |
| --- | --- | --- | --- | --- | --- | --- |
| 1 | 10 x 47 | 2 | 9 months  10 months | Male  Male | Base of a mid-sized tree. | Claw marks visible on tree. |
| 2 | 10 x 20 and 10 x 47 | 2 | 9 months  10 months | Male  Male | Between steps on a ramp leading into a wooden ‘cubby house’. | Area of high activity. |
| 3 | 40 x 30 area made up four boards | 3 | 3 years | Female | Top of a wooden box. | Food used to attract devils. |
| 4 | 30 x 30 square made up of three boards | 4 | 2 years | Female | Base of a large tree | Food used to attract devils. |
| 5 | 7 x 90 and 7 x 90 | 4 | 2 years | Female | Around the base of small tree | Food used to attract devils. |
| 6 | 7 x 23; 7 x 23 and 10 x 23 | 5 | 2 years | Male | Between steps on a ramp leading into a wooden ‘cubby house’. | Area of high activity. |
| 7 | 10 x 70 and 20 x 45 | 7 | 2 years | Male | Base and trunk of large tree | Food used to attract devils. |
| 8 | 10 x 72 | 8 | 3 years | Male | Across horizontal branches | Food used to attract devils. |

**Supplementary Figure 3 | Claw-marked boards resulting from *Sarcophilus harrisii* actualistic trial.** (**A–B**) Station 1. (**C–D**) Station 2. (**E–F**) Station 3. (**G–H**) Station 4. **B, D, F** and **H** have been overlaid with the scratch sets indicated in Supplementary Table 5.


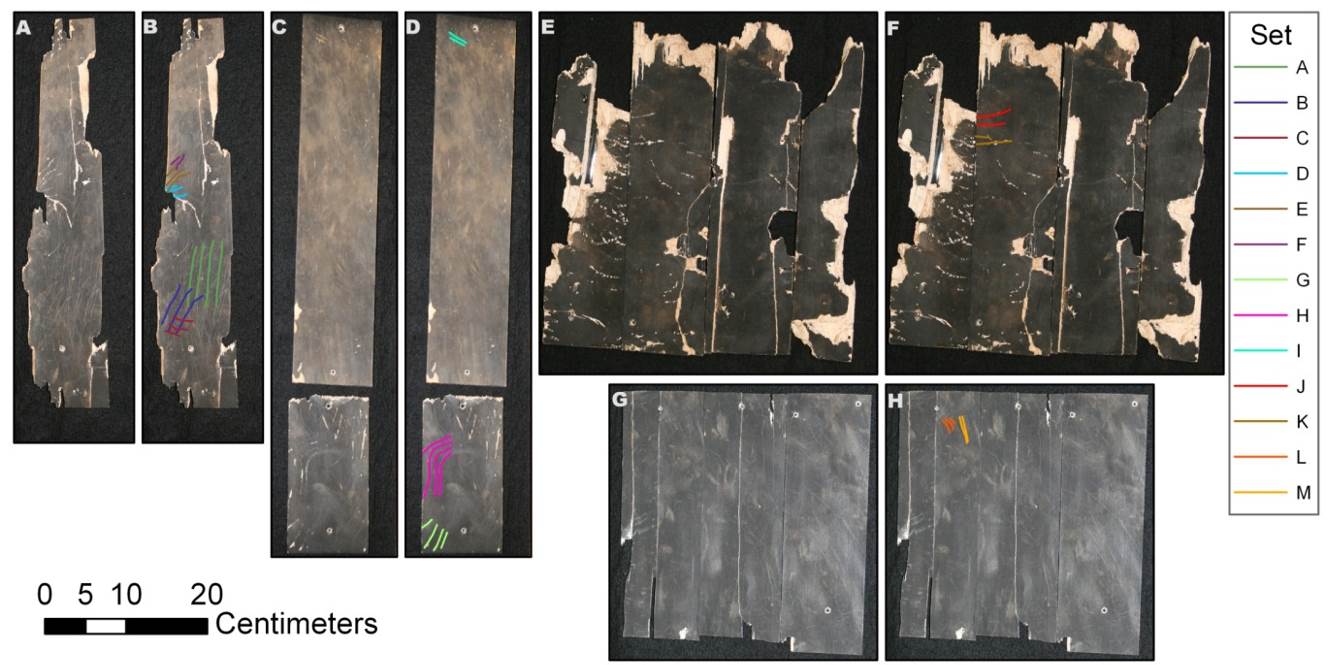


**Supplementary Table 5 | Claw-mark data from *Sarcophilus harrisii* actualistic scratching trial.** Description refers to how the markings were categorised (see Supplementary Table 2). P=parallel, D=diverge, C=converge, DU= diverge upper, DC=diverge centre, DL= diverge lower, CU= converge upper CC= converge centre, CL=converge lower. U, C and L = uppermost, centre and lowermost points. Empty cells indicate where measurements could not be taken due to marks being offset from one another. All measurements in mm.

| **Set** | **No. of marks** | **Mean mark width** | **Length** | **Orientation** | **Inter-Digital Spacing**  **I–II** | | | **Description** | **Inter-Digital Spacing**  **II–III** | | | **Description** | **Inter-Digital Spacing**  **III–IV** | | | **Description** |
| --- | --- | --- | --- | --- | --- | --- | --- | --- | --- | --- | --- | --- | --- | --- | --- | --- |
|  |  |  |  |  | U | C | L |  | U | C | L |  | U | C | L |  |
| A | 4 | 0.7 | 100 | 10 | 11.1 | 12.1 | 11.4 | P | 10.6 | 9.3 | 12.3 | DL | 7.9 | 8.9 | 8.6 | P |
| B | 3 | 0.7 | 50 | 20 | 12.5 | 10.4 | 8.7 | C | 9.9 | 9.8 | 8.5 | P |  |  |  |  |
| C | 3 | 0.5 | 20 | 70 | 10.4 | 10.7 | 10.0 | P | 9.0 | 6.8 | 9.2 | CC |  |  |  |  |
| D | 3 | 1.2 | 15 | 80 | 8.1 | 7.4 | 6.7 | P | 3.5 | 3.6 | 3.7 | P |  |  |  |  |
| E | 3 | 1.6 | 30 | 50 | 6.2 | 4.7 | 3.2 | C | 5.7 | 5.6 | 6.2 | P |  |  |  |  |
| F | 2 | 1.3 | 15 | 50 | 4.0 | 4.2 | 5.0 | P |  |  |  |  |  |  |  |  |
| G | 4 | 1.2 | 30 | 10 | 6.7 | 10.3 | 12.6 | DL | 9.3 | 8.7 | 7.8 | P | 4.9 | 5.6 | 5.0 | P |
| H | 4 | 12 | 60 | 0 | 4.7 | 8.3 | 5.3 | DC | 5.1 | 6.1 | 10.7 | DL | 5.8 | 5.6 | 7.4 | P |
| I | 2 | 1.3 | 20 | -70 | 2.3 | 3.5 | 3.3 | P |  |  |  |  |  |  |  |  |
| J | 2 | 1.6 | 30 | 80 | 13.3 | 8.7 | 8.7 | DU |  |  |  |  |  |  |  |  |
| K | 2 | 1.5 | 15 | 80 | 3.7 | 6.0 | 7.8 | DU |  |  |  |  |  |  |  |  |
| L | 3 | 0.7 | 15 | -40 | 3.1 | 4.1 | 5.2 | D | 3.3 | 4.0 | 4.7 | P |  |  |  |  |
| M | 2 | 0.7 | 30 | -30 | 2.9 | 2.2 | 1.0 | P |  |  |  |  |  |  |  |  |
| N | 2 | 0.6 | 15 | -10 | 4.8 | 4.6 | 4.6 | P |  |  |  |  |  |  |  |  |
| O | 4 | 0.7 | 5 | -60 | 5.7 | 5.7 | 5.9 | P | 4.8 | - | - | P | 4.7 | 4.8 | 4.9 | P |
| P | 4 | 1.3 | 30 | -40 | 7.3 | 6.2 | 7.8 | P | 7.7 | 9.0 | 8.6 | P | 4.8 | 5.3 | 5.1 | P |
| Q | 2 | 0.7 | 35 | 10 | 8.4 | 8.3 | 7.0 | P |  |  |  |  |  |  |  |  |
| R | 2 | 0.8 | 35 | 0 | 5.8 | 5.1 | 2.4 | C |  |  |  |  |  |  |  |  |

*Lasiorhinus latifrons—*Claw marks were distinctly U-shaped in cross section (Supplementary Fig. 4). Measurements are presented in Supplementary Table 6. The distribution of *L. latifrons* IDS appeared approximately normal, which was supported by the Wilk–Shapiro test which did not refute the null hypothesis of a normal distribution (*p =* 0.056). Set length also appeared normally distributed in histogram, and the Wilk–Shapiro test again did not refute the null hypothesis of a normal distribution (*p =* 0.232), though number of replicates (n = 6) is low. The calculations revealed parallel to be the mode variety of marks indicated (four of nine), with the remainder diverging or converging at the centre.

**Supplementary Figure 4 | Claw marks recorded in the *Lasiorhinus latifrons* actualistic trial.** (**A–B**) Station 1. (**C–D**) Station 2. (**E–F**) Station 3. (**G–H**) Station 4. **B, D, F** and **H** have been overlaid with the scratch sets indicated in Supplementary Table 6. Scale bar = 4 cm.


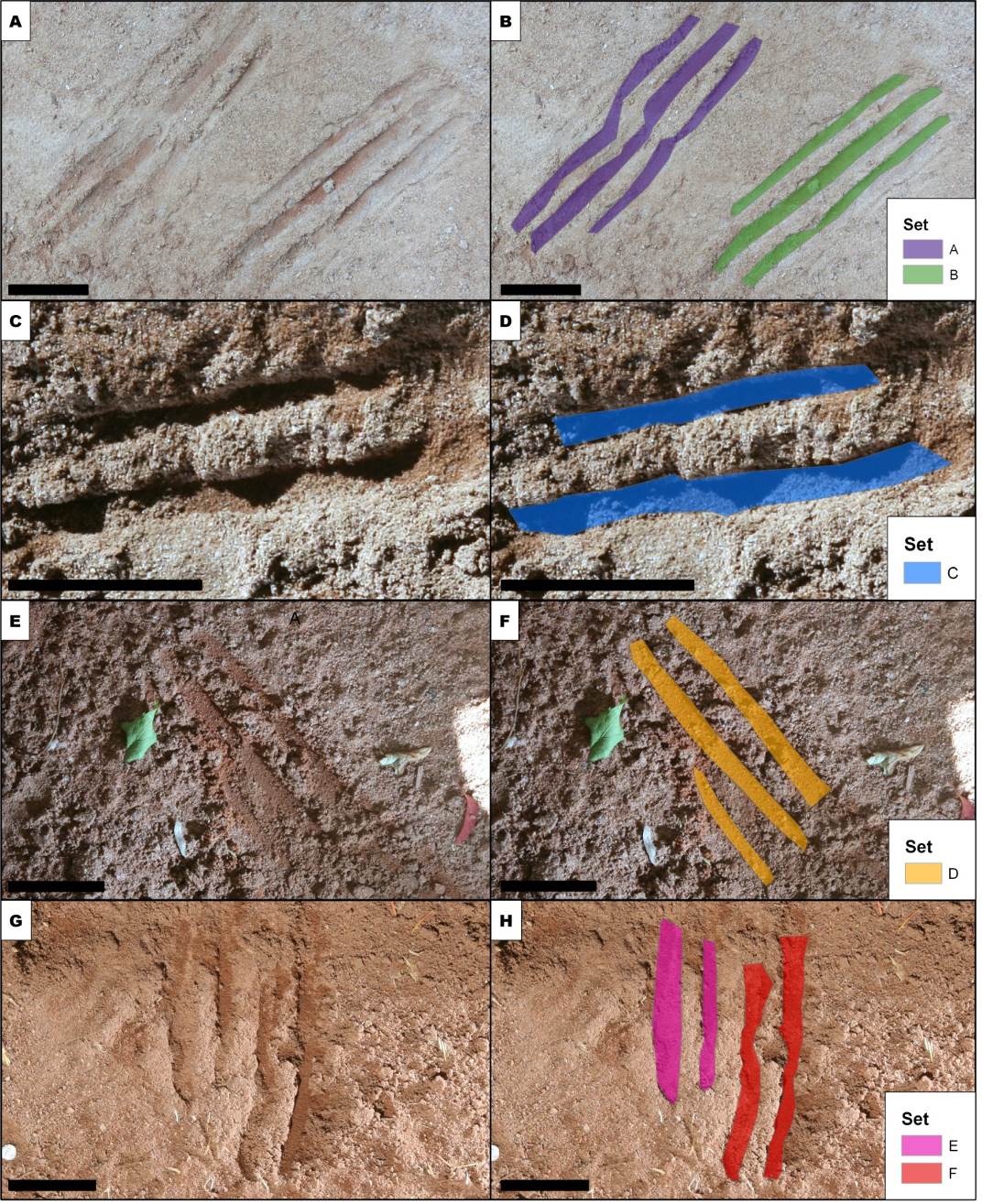


**Supplementary Table 6 | Claw-mark data from *Lasiorhinus latifrons* actualistic scratching trial.** All measurements in mm.

| **Set** | **No. of marks** | **Side** | **Length** | **Mean scratch width** | **Inter-Digital Spacing I–II** | | | **Description** | **Inter-Digital Spacing II–III** | | | **Description** |
| --- | --- | --- | --- | --- | --- | --- | --- | --- | --- | --- | --- | --- |
|  |  |  |  |  | **U** | **C** | **L** |  | **U** | **C** | **L** |  |
| A | 3 | left | 190 | 8 | 10 | 7 | 12 | CC | 10 | 12 | 12 | P |
| B | 3 | right | 180 | 7 | 9 | 12 | 9 | DC | 12 | 12 | 13 | P |
| C | 2 | - | 130 | 8 | 15 | 14 | 11 | CL |  |  |  |  |
| D | 3 | left | 130 | 8 | 12 | 15 | 10 | DC | 10 | 7 | 12 | CC |
| E | 2 | - | 100 | 10 | 12 | 12 | 12 | P |  |  |  |  |
| F | 2 | - | 130 | 11 | 11 | 13 | 14 | P |  |  |  |  |

*Trichosurus vulpecula* and *Phascolarctos cinereus—*Claw-marked bark attributed to these two species was identified in situ and on shed bark picked up off of the ground (Supplementary Fig. 5). Inter-digital spacing for sets attributed to *T. vulpecula* (Supplementary Table 5) appeared approximately normal in histogram, and a Shapiro–Wilk test could not refute the null hypothesis of normal distribution (*p =* 0.710). Only two sets being attributed to *T. vulpecula* precludes detailed statistical analysis of set length. Although both sets were short (≤ 15 mm), many other scratches not in sets thought to be derived from this species were not similarly short so this was not considered a definitive feature. Both sets were also classified as parallel. Sets attributed to *P. cinereus* also included two sets of a distinct morphology from the remaining marks. As well as the short marks often found, when a climbing individual slips it can leave longer marks (Triggs 2004). It is thought that this is the likely explanation for these sets (Supplementary Table 6 I, J). This hypothesis is supported by these marks being associated as well as their being on comparable angles. The IDS histogram appeared approximately normal with a slight positive skew, which is supported by the Shapiro–Wilk test which could not refute a normal distribution (*p =* 0.104). The set length histogram appears to be tri-modal, however this is largely due to low numbers (n = 10) and the binning of values into 5 mm increments. The Shapiro-Wilk test for set length again could not refute a normal distribution (*p =* 0.104). *P. cinereus* attributed scratches also showed a large range in IDS (4.22–15.77 mm, Supplementary Table 6), perhaps reflecting the foot structure of *P. cinereus* where the first two toes are divergent to the remaining three (Triggs 2004). This may also explain only eight of fifteen scratches were classified as parallel, however, no other trends were evident.

**Supplementary Figure 5 | Opportunistically-collected bark claw marked by *Trichosurus vulpecula* and *Phascolarctos cinereus*.** (**A**) Marks attributed to *T. vulpecula*. (**B–C**) Marks attributed to *P. cinereus*. (**D**) Marks attributed to *P. cinereus* slipping. (**E**) Marks attributed to *P. cinereus* demonstrating divergence. Scale bars = 20mm; scale card in **C** = 100 mm.


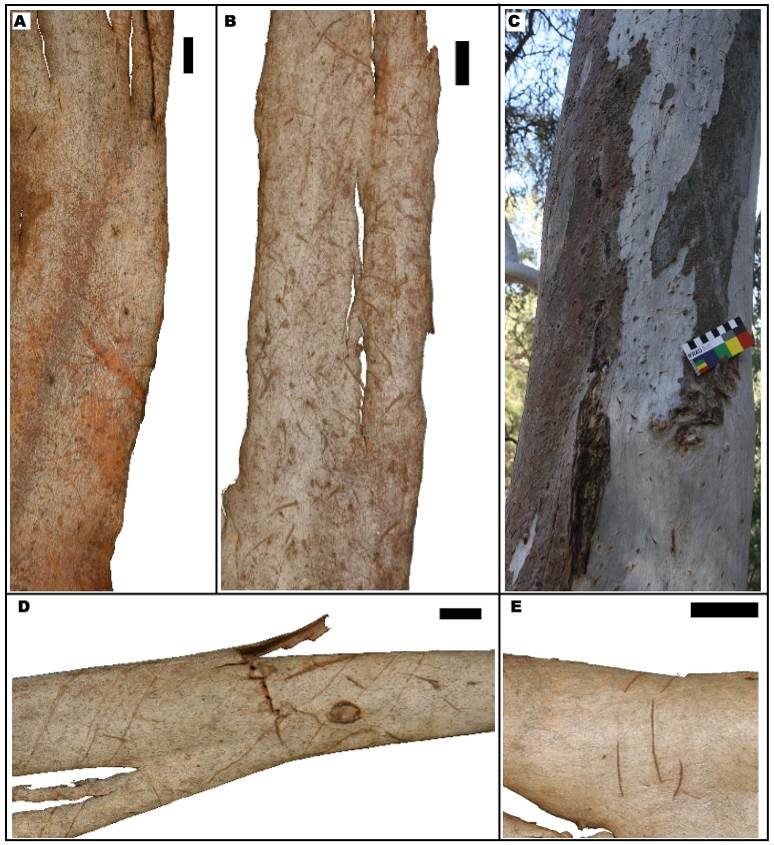


**Supplementary Table 7 | Claw-mark data for *Trichosurus vulpecula*. All measurements in mm.**

| **Set** | **No. of marks** | **Mean mark width** | **Length** | **Inter-Digital Spacing I–III** | | | **Description** | **Inter-Digital Spacing II–III** | | | **Description** |
| --- | --- | --- | --- | --- | --- | --- | --- | --- | --- | --- | --- |
|  |  |  |  | **U** | **C** | **L** |  | **U** | **C** | **L** |  |
| A | 3 | 0.6 | 10 | 3.7 | 3.6 | 2.7 | Parallel | 5.2 | 4.4 | 3.6 | Parallel |
| B | 3 | 0.4 | 15 | 4.1 | 4.4 | 4.2 | Parallel | 3.7 | 3.9 | 4.4 | Parallel |

**Supplementary Table 8 | Claw-mark data for *Phascolarctos cinereus*. All measurements in mm.**

| **Set** | **No. of marks** | **Mean mark width** | **Length** | **Inter-Digital Spacing I–II** | | | **Description** | **Inter-Digital Spacing II–III** | | | **Description** | **Inter-Digital Spacing III–IV** | | | **Description** | **Inter-Digital Spacing IV–V** | | | **Description** |
| --- | --- | --- | --- | --- | --- | --- | --- | --- | --- | --- | --- | --- | --- | --- | --- | --- | --- | --- | --- |
|  |  |  |  | **U** | **C** | **L** |  | **U** | **C** | **L** |  | **U** | **C** | **L** |  | **U** | **C** | **L** |  |
| A | 3 | 0.6 | 30 | 11.4 | 9.4 | 8.7 | D | 7.5 | 9.2 | 11.2 | D |  |  |  |  |  |  |  |  |
| B | 4 | 0.5 | 10 | 4.6 | 4.2 | 4.4 | P | 5.8 | 5.4 | 5.3 | P | 10.5 | 11.3 | 11.7 | P |  |  |  |  |
| C | 4 | 1.0 | 45 | 5.5 | 4.6 | 5.4 | P | 8.3 | 8.1 | 8.4 | P | 4.5 | 6.9 | 10.3 | D |  |  |  |  |
| D | 2 | 0.9 | 30 | 13.6 | 12.5 | 10.1 | CL |  |  |  |  |  |  |  |  |  |  |  |  |
| E | 3 | 0.7 | 30 | 7.6 | 5.9 | 11.8 | DL | 13.0 | 12.7 | 11.6 | P |  |  |  |  |  |  |  |  |
| F | 2 | 0.8 | 25 | 5.7 | 7.7 | 12.1 | DL |  |  |  |  |  |  |  |  |  |  |  |  |
| G | 5 | 0.8 | 10 | 9.6 | 5.3 | 6.9 | CU | 7.1 | 6.1 | 6.1 | P | 15.8 | 13.5 | 12.1 | C | 6.8 | 7.8 | 8.7 | P |
| H | 4 | 0.8 | 45 | 5.6 | 7.6 | 7.2 | DU | 11.6 | 8.8 | 9.4 | CU | 9.9 | 8.9 | 7.0 | C |  |  |  |  |
| I | 3 | 1.0 | >150 | 10.5 | 20.2 | 21.6 | DU | 15.0 | 12.2 | 13.1 | CU |  |  |  |  |  |  |  |  |
| J | 3 | 1.0 | >75 | 27.4 | 13.2 | 17.8 | CU | 0.0 | 15.6 | 11.0 | DU |  |  |  |  |  |  |  |  |

*Thylacoleo carnifex––*Data for claw marks produced in the mock actualistic trial are presented in Supplementary Table 9.

**Supplementary Figure 6 | Mock claw marks of *Thylacoleo carnifex* in modelling clay.** (**A**) With digit I aligned with digit II. (**B**) With digit I extended away from digit II. Scale is 20 mm.

**A
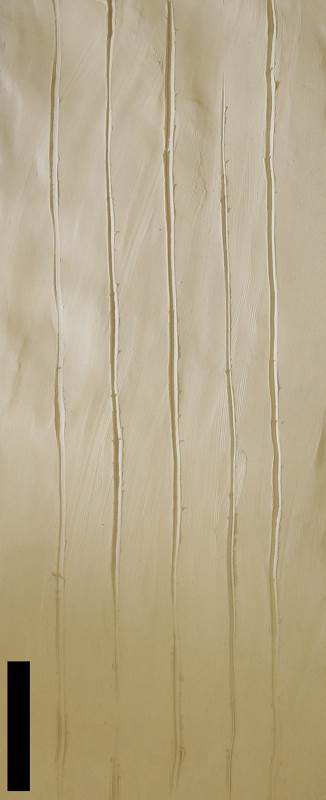
 B
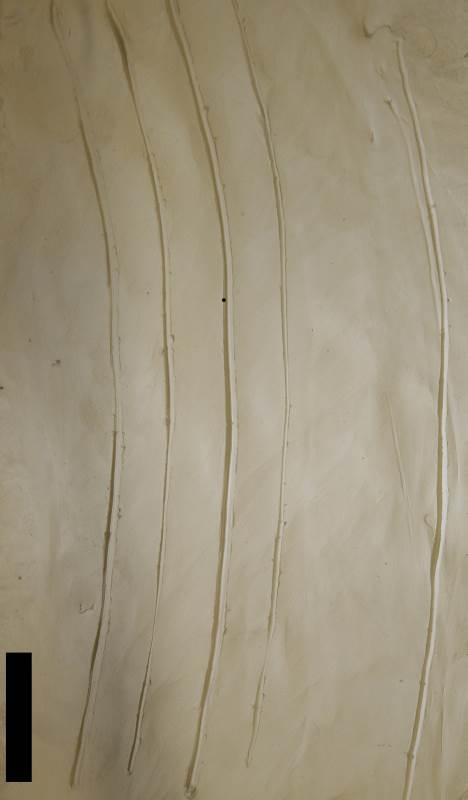
**

**Supplementary Table 9 | Claw-mark measurements from the *Thylacoleo carnifex* mock actualistic trial.** All measurements in mm.

| **Setup** | **Claw Mark Width** | | | | | **Inter-digital Spacing** | | | |
| --- | --- | --- | --- | --- | --- | --- | --- | --- | --- |
|  | **I** | **II** | **III** | **IV** | **V** | **I–II** | **II–III** | **III–IV** | **IV–V** |
| *Thylacoleo carnifex* digit I extended | 2.9 | 1.6 | 2.9 | 2.4 | 2.5 | 15 | 19 | 19 | 19 |
| *Thylacoleo carnifex* digit I flexed | 3.0 | 1.5 | 2.6 | 2.1 | 2.9 | 60 | 17 | 19 | 18 |

*Thylacinus cynocephalus* and *Petrogale xanthopus*––Data collected is contained in Supplementary Table 10.

**Supplementary Table 10 | *Thylacinus cynocephalus* and *Petrogale xanthopus* claw-mark data.** All measurements in mm.

| **Species** | **Side** | **Digit Width** | | | | | **Inter-digital Spacing** | | | |
| --- | --- | --- | --- | --- | --- | --- | --- | --- | --- | --- |
|  |  | **I** | **II** | **III** | **IV** | **V** | **I–II** | **II–III** | **III–IV** | **IV–V** |
| *T. cynocephalus* | Right | 5.40 | 6.24 | 6.61 | 5.56 | 4.88 | 6.80 | 17.95 | 11.30 | 9.45 |
| *P. xanthopus* | Left | 2.56 | 2.92 | 3.31 | 3.21 | 2.41 | 10.10 | 7.81 | 10.53 | 7.97 |
| *P. xanthopus* | Right | 2.34 | 2.17 | 2.47 | 3.14 | 2.74 | 13.76 | 9.45 | 5.97 | 4.53 |

**Trace-fossil Analyses**

Six major scratch-marked areas were identified: Cave Wall South (n = 3); Boulder (n = 41); Central Rock Pile (CRP) North (n = 25), CRP South (n = 26), CRP West (n = 6); Top of CRP (n = 2). Most sets (68.0%) were made up of pairs of marks, with sets of three (22.3%) and four (9.7%) comprising the remainder (Tables S9–S10). Multiple photographs were taken of each area within Tight Entrance Cave, with lighting placed in different positions to highlight scratches, resulting in multiple photographs for each scratch set. Each of these was viewed to identify sets, though this documentation serves to quantify the marks rather than identify all marks present. Exemplars are presented here (Figs 7–29).

**Supplementary Table 11 | Summary of Tight Entrance Cave trace-fossil scratch marks.** Description refers to how marks were categorised (see Supplementary Table 2). Length refers to the average length of the set. CWS = Cave wall south; CRP = Central rock pile. All measurements in mm.

| **Location** | **Sub-Region** | **Set** | **No. of marks** | **Length** | **Angle** | **Inter-digital Spacing I–II** | | | **Description** | **Inter-digital Spacing II–III** | | | **Description** | **Inter-digital Spacing III–IV** | | | **Description** |
| --- | --- | --- | --- | --- | --- | --- | --- | --- | --- | --- | --- | --- | --- | --- | --- | --- | --- |
|  |  |  |  |  |  |  |  |  |  |  |  |  |  |  |  |  |  |
|  |  |  |  |  |  | **U** | **C** | **L** |  | **U** | **C** | **L** |  | **U** | **C** | **L** |  |
| Cave Wall South | - | A | 3 | 30 | 35 | 7 | 8 | 10 | D | 7 | 8 | 10 | D |  |  |  |  |
| Cave Wall South | - | B | 2 | 72 | -50 | 25 | 20 | 16 | C |  |  |  |  |  |  |  |  |
| Cave Wall South | - | C | 3 | 10 | 13 | 11 | 10 | 9 | P | 10 | 9 | 7 | C |  |  |  |  |
| Boulder | 1 | D | 2 | 35 | -10 | 7 | 7 | 8 | P |  |  |  |  |  |  |  |  |
| Boulder | 1 | E | 2 | 55 | -56 | 10 | 8 | 9 | P |  |  |  |  |  |  |  |  |
| Boulder | 1 | F | 3 | 33 | 6 | 12 | 14 | 13 | P | 15 | 15 | 14 | P |  |  |  |  |
| Boulder | 1 | G | 2 | 40 | 27 | 11 | 13 | 12 | P |  |  |  |  |  |  |  |  |
| Boulder | 2 | H | 2 | 40 | 33 | 5 | 8 | 7 | DU |  |  |  |  |  |  |  |  |
| Boulder | 2 | I | 2 | 150 | 1 | 14 | 14 | 13 | P |  |  |  |  |  |  |  |  |
| Boulder | 2 | J | 2 | 120 | 31 | 20 | 11 | 16 | CC |  |  |  |  |  |  |  |  |
| Boulder | 2 | K | 2 | 90 | -69 | 21 | 20 | 19 | P |  |  |  |  |  |  |  |  |
| Boulder | 2 | L | 2 | 50 | 8 | 17 | 18 | 17 | P |  |  |  |  |  |  |  |  |
| Boulder | 2 | M | 2 | 80 | 46 | 11 | 14 | 23 | D |  |  |  |  |  |  |  |  |
| Boulder | 2 | N | 2 | 70 | -67 | 16 | 16 | 17 | P |  |  |  |  |  |  |  |  |
| Boulder | 2 | O | 2 | 75 | -15 | 13 | 16 | 18 | DU |  |  |  |  |  |  |  |  |
| Boulder | 2 | P | 2 | 40 | -39 | 4 | 6 | 6 | P |  |  |  |  |  |  |  |  |
| Boulder | 2 | Q | 2 | 50 | -30 | 28 | 19 | 14 | C |  |  |  |  |  |  |  |  |
| Boulder | 2 | R | 2 | 100 | 33 | 4 | 5 | 13 | DL |  |  |  |  |  |  |  |  |
| Boulder | 2 | S | 2 | 30 | 40 | 15 | 14 | 12 | C |  |  |  |  |  |  |  |  |
| Boulder | 2 | T | 2 | 55 | 33 | 16 | 15 | 8 | CL |  |  |  |  |  |  |  |  |
| Boulder | 3 | U | 2 | 50 | -67 | 16 | 10 | 12 | CU |  |  |  |  |  |  |  |  |
| Boulder | 3 | V | 2 | 30 | -19 | 5 | 6 | 5 | P |  |  |  |  |  |  |  |  |
| Boulder | 3 | W | 2 | 40 | -4 | 7 | 7 | 4 | CL |  |  |  |  |  |  |  |  |
| Boulder | 4 | X | 2 | 55 | -3 | 16 | 14 | 11 | CL |  |  |  |  |  |  |  |  |
| Boulder | 4 | Y | 3 | 35 | 29 | 9 | 8 | 5 | CL | 6 | 7 | 6 | P |  |  |  |  |
| Boulder | 5 | Z | 2 | 35 | 42 | 9 | 7 | 8 | P |  |  |  |  |  |  |  |  |
| Boulder | 5 | AA | 3 | 53 | 60 | 4 | 9 | 8 | DU | 11 | 11 | 9 | P |  |  |  |  |
| Boulder | 6 | AB | 2 | 35 | -52 | 12 | 11 | 6 | CL |  |  |  |  |  |  |  |  |
| Boulder | 6 | AC | 4 | 17 | -63 | 12 | 12 | 11 | P | 8 | 9 | 10 | P | 8 | 7 | 7 | P |
| Boulder | 6 | AD | 3 | 17 | -57 | 7 | 7 | 7 | P | 16 | 14 | 9 | CL |  |  |  |  |
| Boulder | 6 | AE | 3 | 22 | 32 | 9 | 8 | 8 | P | 12 | 13 | 11 | P |  |  |  |  |
| Boulder | 6 | AF | 2 | 35 | -88 | 12 | 13 | 13 | P |  |  |  |  |  |  |  |  |
| Boulder | 6 | AG | 2 | 65 | 83 | 8 | 10 | 8 | P |  |  |  |  |  |  |  |  |
| Boulder | 6 | AH | 2 | 30 | 2 | 12 | 11 | 11 | P |  |  |  |  |  |  |  |  |
| Boulder | 6 | AI | 3 | 75 | -45 | 32 | 28 | 31 | CC | 11 | 19 | 7 | DC |  |  |  |  |
| Boulder | 6 | AJ | 3 | 27 | -43 | 5 | 5 | 7 | P | 4 | 9 | 9 | DU |  |  |  |  |
| Boulder | 6 | AK | 2 | 80 | 38 | 10 | 14 | 9 | DC |  |  |  |  |  |  |  |  |
| Boulder | 7 | AL | 3 | 24 | 43 | 10 | 13 | 13 | DU | 9 | 11 | 11 | P |  |  |  |  |
| Boulder | 7 | AM | 2 | 50 | 36 | 6 | 9 | 15 | D |  |  |  |  |  |  |  |  |
| Boulder | 7 | AN | 4 | 35 | -68 | 7 | 8 | 7 | P | 5 | 6 | 7 | P | 9 | 10 | 7 | CL |
| Boulder | 7 | AO | 2 | 50 | -68 | 11 | 13 | 12 | P |  |  |  |  |  |  |  |  |
| Boulder | 7 | AP | 3 | 50 | -10 | 18 | 21 | 25 | D | 15 | 15 | 15 | P |  |  |  |  |
| Boulder | 8 | AQ | 2 | 70 | 22 | 16 | 18 | 30 | DL |  |  |  |  |  |  |  |  |
| Boulder | 8 | AR | 2 | 65 | -35 | 17 | 15 | 13 | C |  |  |  |  |  |  |  |  |
| CRP West | - | AS | 4 | 81 | 73 | 13 | 13 | 13 | P | 11 | 8 | 7 | CU | 9 | 8 | 6 | C |
| CRP West | - | AT | 2 | 30 | -26 | 17 | 18 | 15 | CL |  |  |  |  |  |  |  |  |
| CRP West | - | AU | 2 | 37 | 68 | 17 | 17 | 14 | CL |  |  |  |  |  |  |  |  |
| CRP West | - | AV | 2 | 50 | -33 | 14 | 11 | 9 | DU |  |  |  |  |  |  |  |  |
| CRP West | - | AW | 2 | 40 | 12 | 11 | 12 | 14 | D |  |  |  |  |  |  |  |  |
| CRP West | - | AX | 2 | 105 | 6 | 18 | 20 | 21 | D |  |  |  |  |  |  |  |  |
| CRP South | 1 | AY | 2 | 75 | 0 | 14 | 15 | 7 | CL |  |  |  |  |  |  |  |  |
| CRP South | 1 | AZ | 2 | 45 | -31 | 23 | 22 | 20 | C |  |  |  |  |  |  |  |  |
| CRP South | 1 | BA | 2 | 30 | 33 | 10 | 8 | 9 | P |  |  |  |  |  |  |  |  |
| CRP South | 2 | BB | 3 | 35 | 4 | 5 | 5 | 6 | P | 6 | 8 | 8 | P |  |  |  |  |
| CRP South | 2 | BC | 3 | 40 | 28 | 13 | 14 | 20 | DL | 17 | 16 | 16 | P |  |  |  |  |
| CRP South | 2 | BD | 2 | 35 | 50 | 13 | 15 | 19 | DL |  |  |  |  |  |  |  |  |
| CRP South | 3 | BE | 2 | 24 | -49 | 9 | 9 | 9 | P |  |  |  |  |  |  |  |  |
| CRP South | 3 | BF | 2 | 35 | 8 | 3 | 3 | 4 | P |  |  |  |  |  |  |  |  |
| CRP South | 3 | BG | 3 | 30 | 46 | 11 | 11 | 12 | P | 11 | 10 | 11 | P |  |  |  |  |
| CRP South | 3 | BH | 2 | 24 | -23 | 6 | 8 | 10 | D |  |  |  |  |  |  |  |  |
| CRP South | 4 | BI | 2 | 40 | 42 | 11 | 9 | 11 | P |  |  |  |  |  |  |  |  |
| CRP South | 4 | BJ | 2 | 35 | 22 | 7 | 4 | 11 | CC |  |  |  |  |  |  |  |  |
| CRP South | 4 | BK | 3 | 30 | -8 | 10 | 7 | 6 | CU | 3 | 11 | 8 | DC |  |  |  |  |
| CRP South | 4 | BL | 2 | 15 | 30 | 9 | 9 | 9 | P |  |  |  |  |  |  |  |  |
| CRP South | 4 | BM | 2 | 5 | 21 | 3 | 4 | 5 | P |  |  |  |  |  |  |  |  |
| CRP South | 5 | BN | 2 | 30 | 27 | 5 | 5 | 4 | P |  |  |  |  |  |  |  |  |
| CRP South | 5 | BO | 2 | 10 | 11 | 7 | 6 | 7 | P |  |  |  |  |  |  |  |  |
| CRP South | 5 | BP | 4 | 16 | 52 | 4 | 4 | 3 | P | 8 | 7 | 8 | P | 8 | 9 | 9 | P |
| CRP South | 5 | BQ | 2 | 25 | 7 | 8 | 9 | 6 | CL |  |  |  |  |  |  |  |  |
| CRP South | 5 | BR | 2 | 45 | 30 | 13 | 12 | 10 | C |  |  |  |  |  |  |  |  |
| CRP South | 5 | BS | 4 | 20 | -12 | 6 | 6 | 4 | P | 3 | 5 | 4 | P | 6 | 4 | 4 | P |
| CRP South | 5 | BT | 2 | 30 | -21 | 6 | 7 | 9 | D |  |  |  |  |  |  |  |  |
| CRP South | 5 | BU | 2 | 12 | -66 | 10 | 11 | 12 | P |  |  |  |  |  |  |  |  |
| CRP South | 5 | BV | 3 | 10 | 56 | 8 | 6 | 5 | C | 4 | 3 | 4 | P |  |  |  |  |
| CRP South | 5 | BW | 4 | 5 | 72 | 3 | 3 | 3 | P | 5 | 5 | 6 | P | 7 | 6 | 6 | P |
| CRP South | 5 | BX | 2 | 35 | 52 | 7 | 10 | 10 | DU |  |  |  |  |  |  |  |  |
| CRP Top | - | BY | 3 | 50 | -60 | 12 | 11 | 10 | P | 13 | 17 | 20 | D |  |  |  |  |
| CRP Top | - | BZ | 3 | 43 | -31 | 15 | 15 | 18 | DL | 12 | 15 | 21 | D |  |  |  |  |
| CRP North | 1 | CA | 2 | 27 | -83 | 8 | 4 | 5 | CU |  |  |  |  |  |  |  |  |
| CRP North | 1 | CB | 2 | 25 | -76 | 9 | 12 | 13 | DU |  |  |  |  |  |  |  |  |
| CRP North | 1 | CC | 3 | 15 | 37 | 3 | 4 | 6 | D | 6 | 7 | 8 | P |  |  |  |  |
| CRP North | 1 | CD | 2 | 20 | 55 | 15 | 16 | 15 | P |  |  |  |  |  |  |  |  |
| CRP North | 2 | CE | 3 | 100 | -24 | 11 | 12 | 13 | P | 11 | 10 | 10 | P |  |  |  |  |
| CRP North |  | - |  |  |  |  |  |  |  | 12 | 17 | 14 | DC |  |  |  |  |
| CRP North | 2 | CF | 4 | 30 | -5 | 9 | 7 | 8 | P | 4 | 6 | 6 | P | 9 | 9 | 8 | P |
| CRP North | 2 | CG | 4 | 5 | 10 | 7 | 7 | 6 | P | 4 | 4 | 4 | P | 7 | 7 | 7 | P |
| CRP North | 2 | CH | 2 | 20 | -84 | 4 | 5 | 6 | P |  |  |  |  |  |  |  |  |
| CRP North | 2 | CI | 2 | 15 | 41 | 3 | 3 | 4 | P |  |  |  |  |  |  |  |  |
| CRP North | 2 | CJ | 2 | 25 | 3 | 5 | 5 | 5 | P |  |  |  |  |  |  |  |  |
| CRP North | 2 | CK | 3 | 25 | -47 | 6 | 6 | 7 | P | 6 | 4 | 5 | P |  |  |  |  |
| CRP North | 2 | CL | 2 | 15 | 81 | 6 | 6 | 7 | P |  |  |  |  |  |  |  |  |
| CRP North | 3 | CM | 4 | 15 | 35 | 14 | 10 | 7 | C | 10 | 9 | 6 | CL | 9 | 8 | 7 | P |
| CRP North | 3 | CN | 2 | 30 | -46 | 10 | 12 | 17 | DL |  |  |  |  |  |  |  |  |
| CRP North | 3 | CO | 2 | 30 | -50 | 7 | 7 | 7 | P |  |  |  |  |  |  |  |  |
| CRP North | 3 | CP | 2 | 35 | 35 | 7 | 8 | 7 | P |  |  |  |  |  |  |  |  |
| CRP North | 3 | CQ | 2 | 15 | -34 | 3 | 4 | 7 | DL |  |  |  |  |  |  |  |  |
| CRP North | 3 | CR | 4 | 27 | 81 | 7 | 7 | 6 | P | 14 | 11 | 9 | CU | 6 | 7 | 6 | P |
| CRP North | 3 | CS | 3 | 58 | 35 | 8 | 8 | 12 | DL | 8 | 9 | 10 | P |  |  |  |  |
|  |  | - |  |  |  | 12 | 14 | 11 | CL |  |  |  |  |  |  |  |  |
| CRP North | 3 | CT | 2 | 15 | -46 | 3 | 3 | 3 | P |  |  |  |  |  |  |  |  |
| CRP North | 3 | CU | 3 | 27 | -50 | 4 | 4 | 6 | P | 5 | 8 | 7 | DU |  |  |  |  |
| CRP North | 3 | CV | 2 | 25 | 35 | 13 | 12 | 12 | P |  |  |  |  |  |  |  |  |
| CRP North | 3 | CW | 2 | 20 | -34 | 11 | 9 | 8 | C |  |  |  |  |  |  |  |  |
| CRP North | 4 | CX | 2 | 97 | 35 | 16 | 16 | 24 | DL |  |  |  |  |  |  |  |  |
| CRP North | 4 | CY | 2 | 40 | -34 | 9 | 9 | 12 | DL |  |  |  |  |  |  |  |  |

**Supplementary Table 10 |** Summary of Tight Entrance Cave claw-mark characteristics.

|  | **Parallel** | **Diverge** | **Converge** | **Diverge upper** | **Diverge lower** | **Converge upper** | **Converge lower** | **Diverge centre** | **Converge centre** |
| --- | --- | --- | --- | --- | --- | --- | --- | --- | --- |
| Count | 79 | 12 | 10 | 9 | 10 | 5 | 12 | 4 | 3 |
| % | 54.9 | 8.3 | 6.9 | 6.3 | 6.9 | 3.5 | 8.3 | 2.8 | 2.1 |

*Cave Wall South*—The wall itself is relatively straight in this area with a slight overhang. Its surface is highly irregular and an area of active moonmilk growth. Moonmilk is a crystalline formation of calcite and water, which forms a soft spongy surface in moist, cool caves (Borsate et al. 2000). Active growth of moonmilk is evinced by the precipitated calcite occurring in the TEC excavations between depositional units (Prideaux et al 2010). That the marks are still visible on this dynamic surface suggests a relatively young age for the marks as similar archaeological markings in moonmilk have been obscured by this process (Bednarik 1999). In addition, moonmilk forms without bedding planes or similar and so also establishes that these markings are certainly scratch marks, rather than erosional features. While there are some regions nearby with sediment evident on the walls, much of this is considered to have been “deposited” there during the exuberant activities of the Hatcher excavations of the early 1990s (Prideaux *et al.* 2010). This area only recorded a small number of scratches, included due to their exceptional preservation.

Three isolated scratch sets were noted in this region on the soft limestone wall (Supplementary Fig. 7). Marks are situated 2.1 m above the sediment floor. The precise past height of the floor is uncertain due to lack of data recording during amateur excavations of the bone deposit in the early 1990s, but judging from adjacent in situ sediment it was probably around 1.5 m below the claw marks when they were made. The lack of surrounding marks, the overhanging nature of the wall and lack of ledges indicates that the animal was not climbing when the scratches were made. The orientation of the upper two scratch sets suggests that they may have been made with the left and right forepaws during the same action. This would be consistent with an older individual of *Thylacoleo carnifex* standing on its hind limbs against the wall. An approximated bipedal standing height of 1.5 m would be consistent with published skeletal measurements (Finch & Freedman 1988). The isolation of the marks suggest a one-off occurrence of inquisitive behaviour.

**Supplementary Figure 7 | Claw-marked surface, Cave Wall South, Tight Entrance Cave.** (**A**) Original photograph. (**B**) Identified scratch sets indicated. Scale bar equals 10 cm.

1.
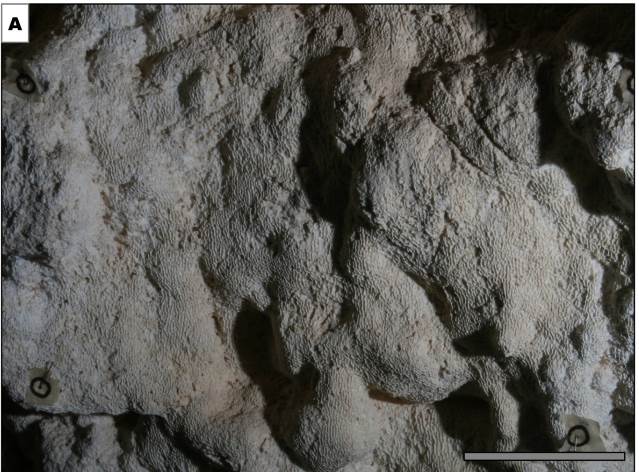

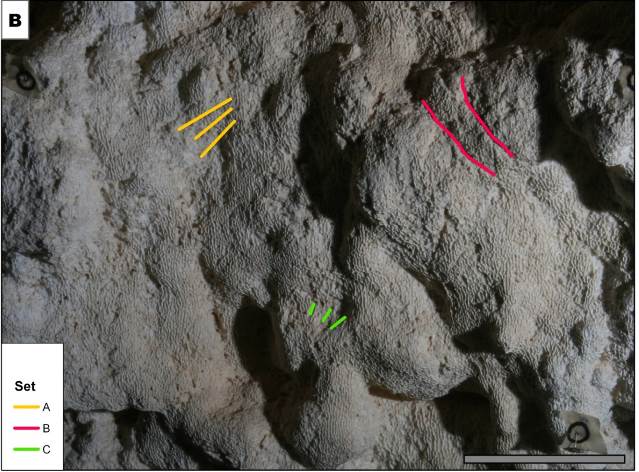


*Boulder—*The exposed portion of this limestone feature is approximately 4 m x 8 m x 3 m. Most of it is buried by sediments. The Boulder was partially undercut at its southeastern end during the Hatcher excavation of the early 1990s (Prideaux *et al.* 2010). The surface varies from white limestone on the scratch-marked surfaces to sediment-covered regions and areas of speleothem growth. Most of the surface is fairly uniform and smooth compared to other surfaces within the cave. Claw marks are abundant but confined to an area on the southeastern face of 4 m x 3 m, which was divided in eight smaller sub-regions for analysis (Supplementary Fig. 8). Marks are generally long and variable in orientation, creating a pattern of diamonds across the surface (Supplementary Figs 9–16). This cross hatching evident also rules out the markings forming along bedding planes or natural faults in limestone through erosion. Due to the heavily marked nature of this area, individual sets are difficult to identify. However, 41 sets were distinguished due to their being deeper or at a slightly different angle to other scratches. The density of claw marks and steepness of the Boulder face suggest that marks were made during purposeful climbing. That individuals chose this steeper route, given that a less steep route to the CRP clearly existed, suggests that this route may simply have been chosen because it was quicker.

**Supplementary Figure 8 | Overview of Boulder sub-regions within which scratch sets were analysed.** Scale bar = 1 m.


| **Supplementary Figure 9 | Claw-marked surface, Boulder sub-region 1.** (**A**) Original photograph. (**B**) Identified scratch sets. Scale bar = 10 cm. | **Supplementary Figure 10 | Claw-marked surface, Boulder sub-region 2.** (**A**) Original photograph. (**B**) Identified scratch sets. Scale bar = 10 cm. |
| --- | --- |
| 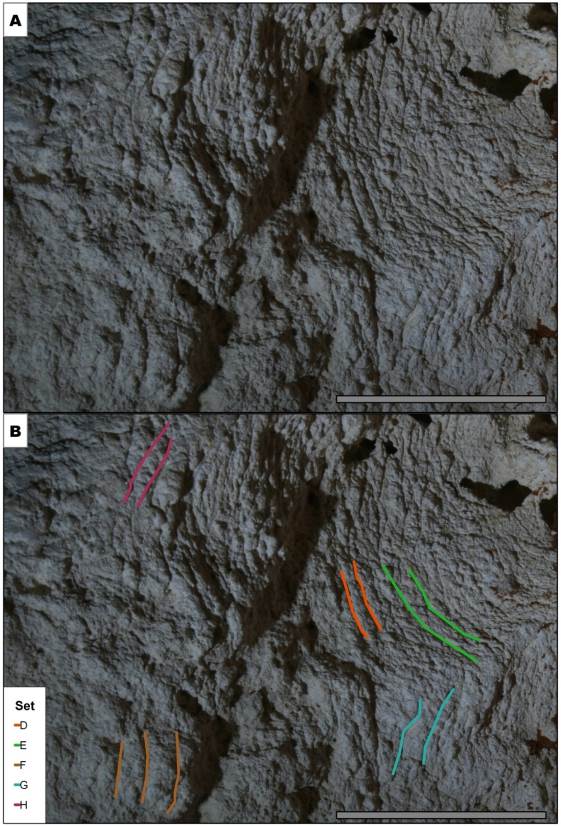 | 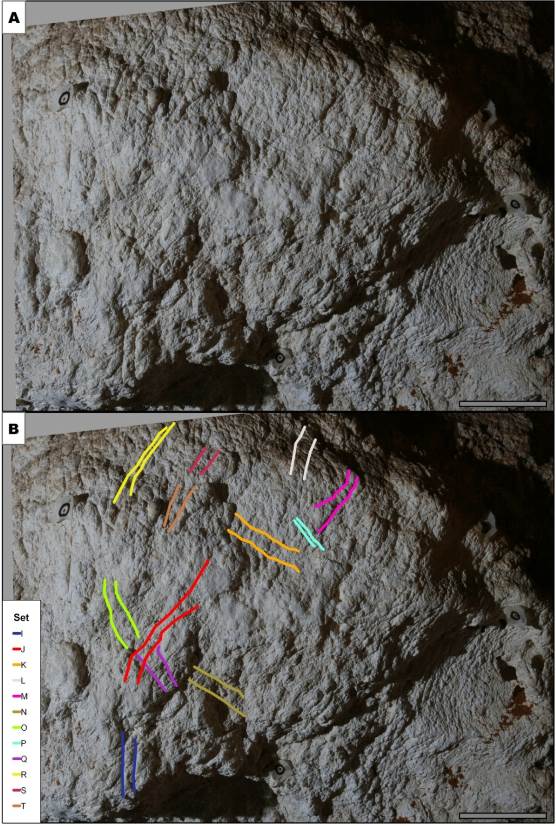 |

| **Supplementary Figure 11 | Claw-marked surface, sub-region 3, southeast face of Boulder.** (**A**) Original photograph. (**B**) Identified scratch sets. Scale bar = 10 cm. | **Supplementary Figure 12 | Claw-marked surface, Boulder sub-region 4.** (**A**) Original photograph. (**B**) Identified scratch sets. Scale bar = 10 cm. |
| --- | --- |
| **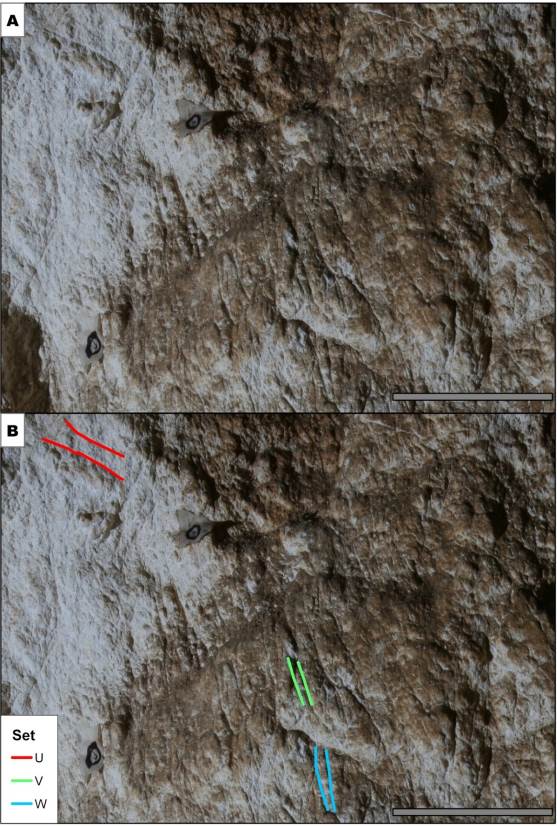** | **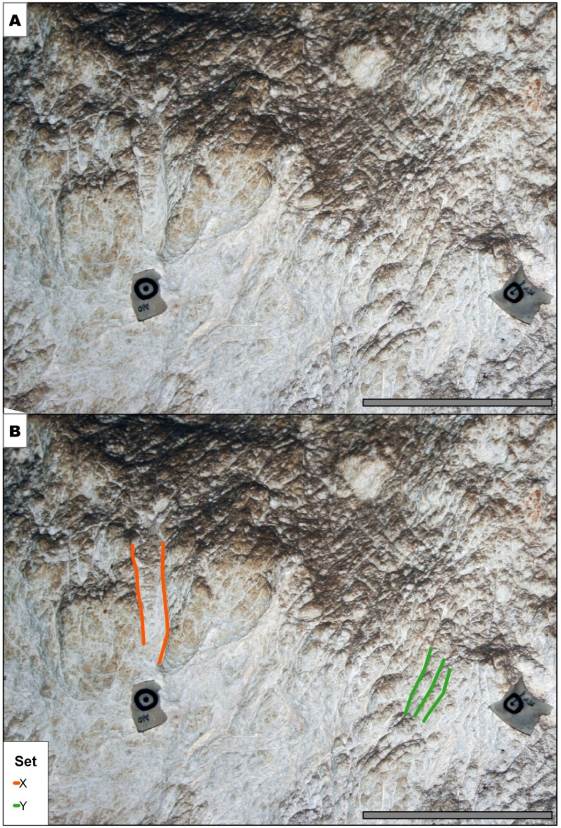** |

| **Supplementary Figure 13 | Claw-marked surface, Boulder sub-region 5.** (**A**) Original photograph. (**B**) Identified scratch sets. Scale bar = 10 cm. | **Supplementary Figure 14 | Claw-marked surface, Boulder sub-region 6.** (**A**) Original photograph. (**B**) Identified scratch sets. Scale bar = 10 cm. |
| --- | --- |
| 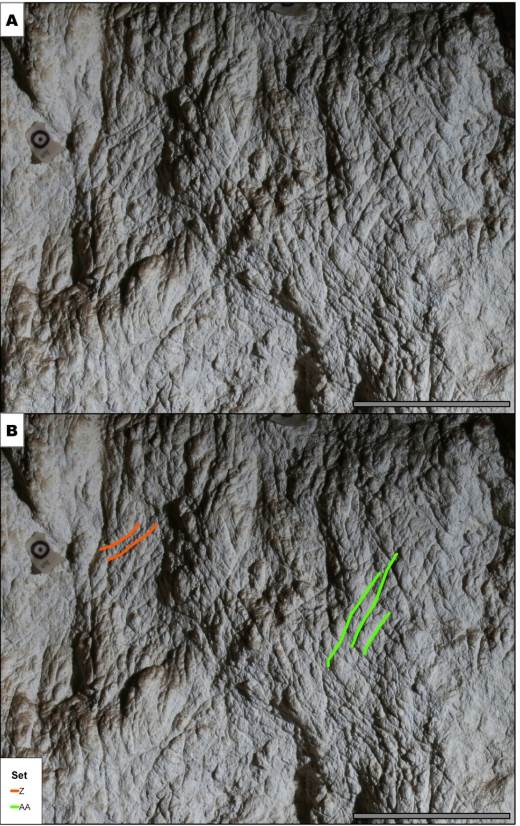 | 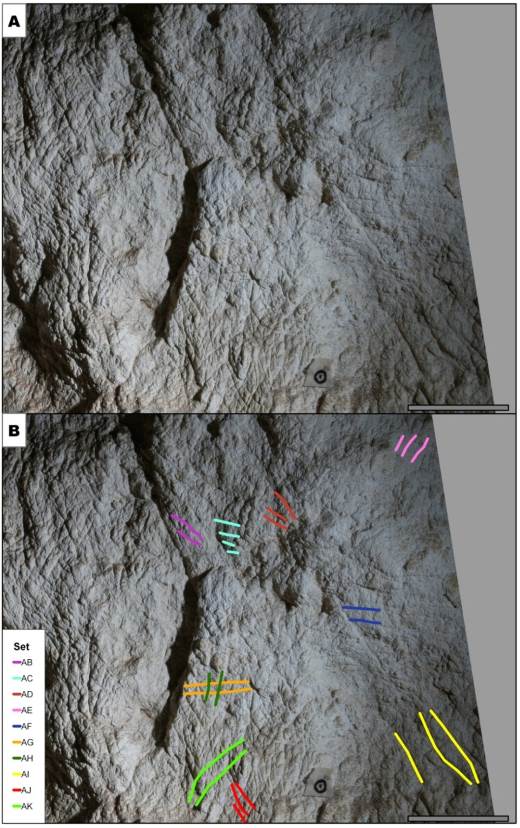 |

| **Supplementary Figure 15 | Claw-marked surface, sub-region 7, southeast face of Boulder.** (**A**) Original photograph. (**B**) Identified scratch sets. Scale bar = 10 cm. | **Supplementary Figure 16 | Claw-marked surface, Boulder sub-region 8.** (**A**) Original photograph. (**B**) Identified scratch sets. Scale bar = 10 cm. |
| --- | --- |
| 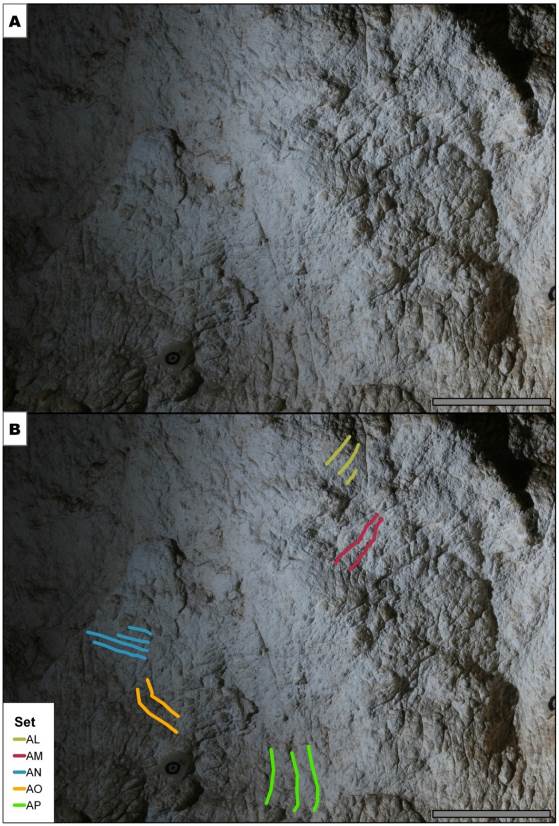 | 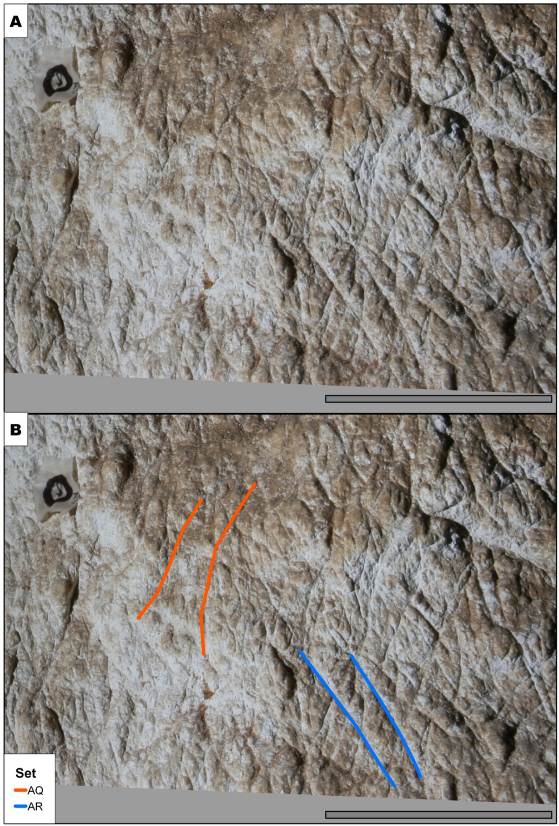 |

*Central Rock Pile—*The CRP dominates the centre of the main chamber of Tight Entrance Cave (Fig. 1). The features was divided into several regions for analysis of scratch sets (Supplementary Fig. 17).

Claw marks on the western face of the CRP (CRP West) are contiguous with the abundant marks on the south face (CRP South), but are located on a separate limestone slab distinct from the remainder of the CRP. CRP West claw marks are less concentrated than in CRP South, but are nevertheless abundant. This region preserved many long linear scratches on the sediment stained limestone, however only six sets could be distinguished (Supplementary Table 11, Supplementary Fig. 18).

CRP South constitutes the largest area of scratches within the cave. The surface is composed of what once was a single boulder that subsequently broke apart and became re-cemented together by sediments, resulting in a semi-continuous, highly variable surface (Supplementary Figs 17, 19–23). Coating sediment thickness varies between sub-regions. Due to the highly-variable surface, preservation of claw marks also varies. The angular nature of many surfaces also impeded the preservation of distinguishable scratch sets, resulting in only 27 sets being identified in CRP South. One area within this region is covered in Karren, which reflects past flowing water, presumably via overhead solution pipes.

**Supplementary Figure 17 | Overview of Central Rock Pile West and South sub-regions within which scratch sets were analysed.** Numbered areas on the right are within CRP south. Scale = 1 m.

1.
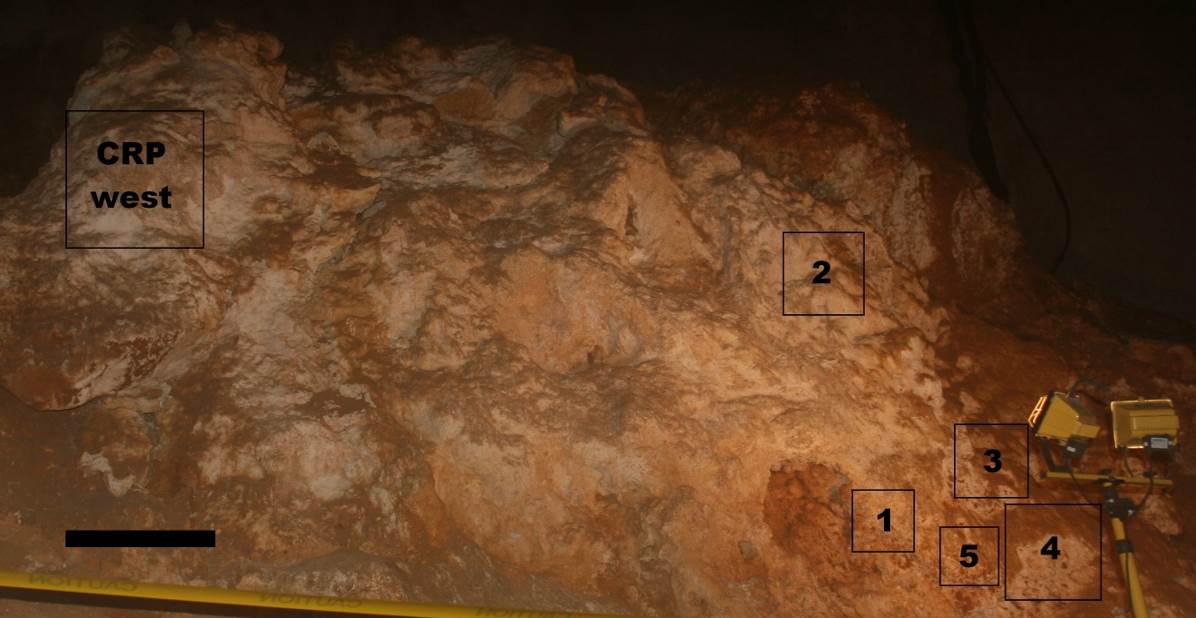


| **Supplementary Figure 18 | Claw-marked surface, CRP West.** (**A**) Original photograph. (**B**) Identified scratch sets. Scale bar = 10 cm. | **Supplementary Figure 19 | Claw-marked surface, CRP South 1.** (**A**) Original photograph. (**B**) Identified scratch sets. Scale bar = 10 cm. |
| --- | --- |
| 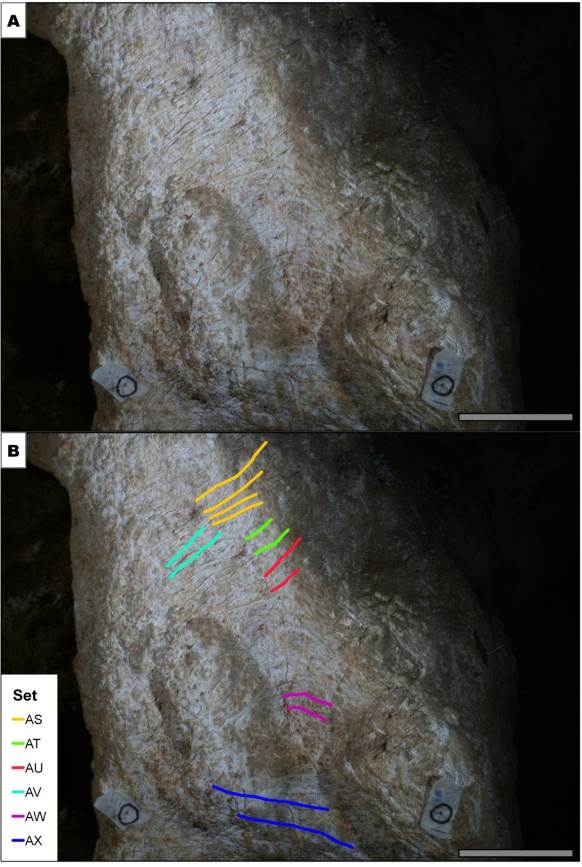 | 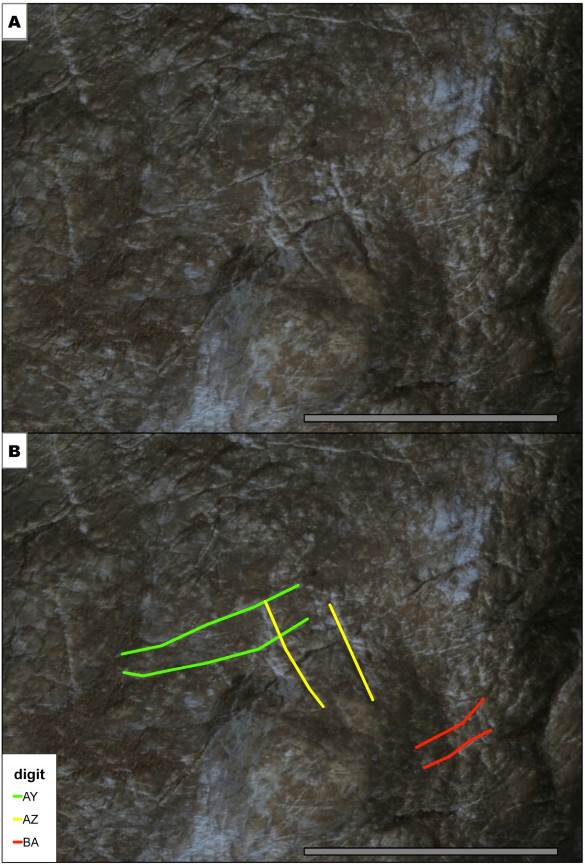 |

| **Supplementary Figure 20 | Claw-marked surface, CRP South 2.** (**A**) Original photograph. (**B**) Identified scratch sets. Scale bar = 10 cm. | **Supplementary Figure 21 | Claw-marked surface, CRP South 3.** (**A**) Original photograph. (**B**) Identified scratch sets. Scale bar = 10 cm. |
| --- | --- |
| 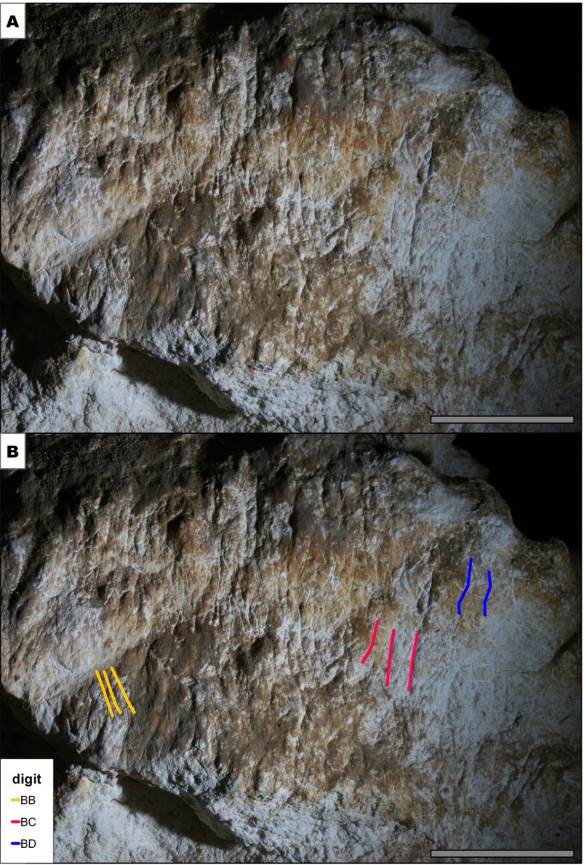 | 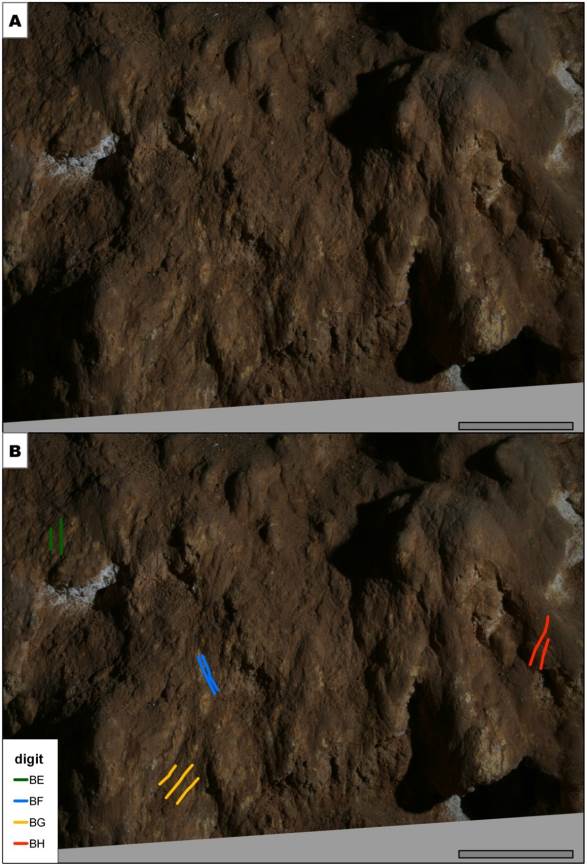 |

| **Supplementary Figure 22 | Claw-marked surface, CRP South 4.** (**A**) Original photograph. (**B**) Identified scratch sets. Scale bar = 10 cm. | **Supplementary Figure 23 | Claw-marked surface, CRP South 5.** (**A**) Original photograph. (**B**) Identified scratch sets. Scale bar = 10 cm. |
| --- | --- |
| 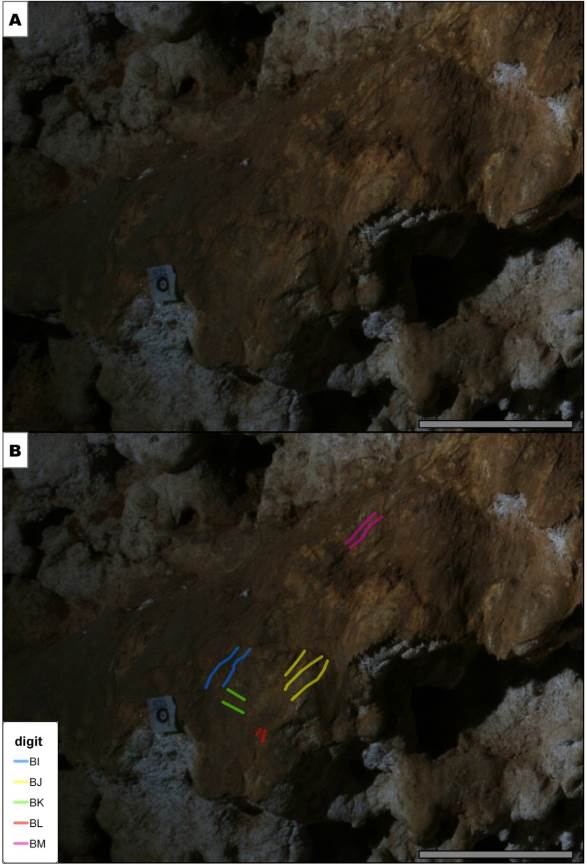 | 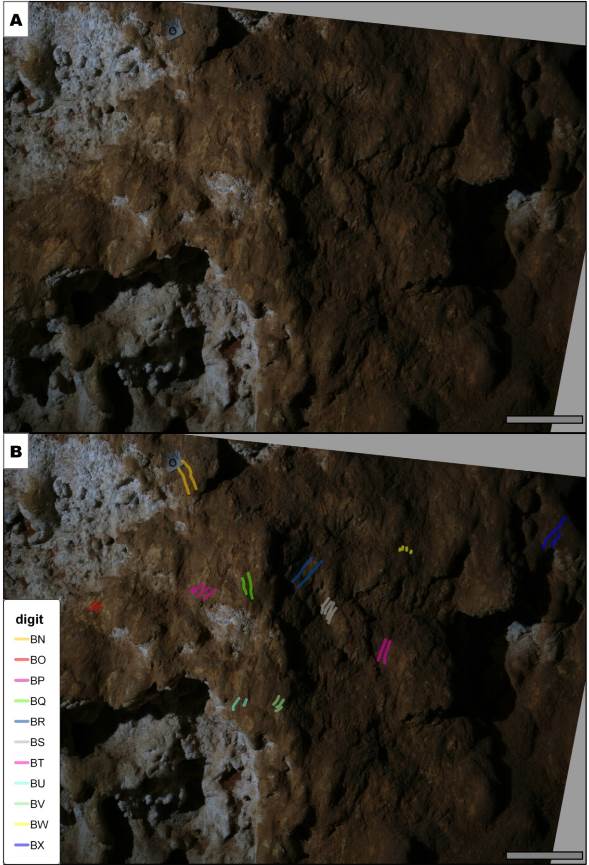 |

**Supplementary Figure 24 | Overview of Central Rock Pile North within which scratch sets were analysed.** Scale = 1 m.

1.
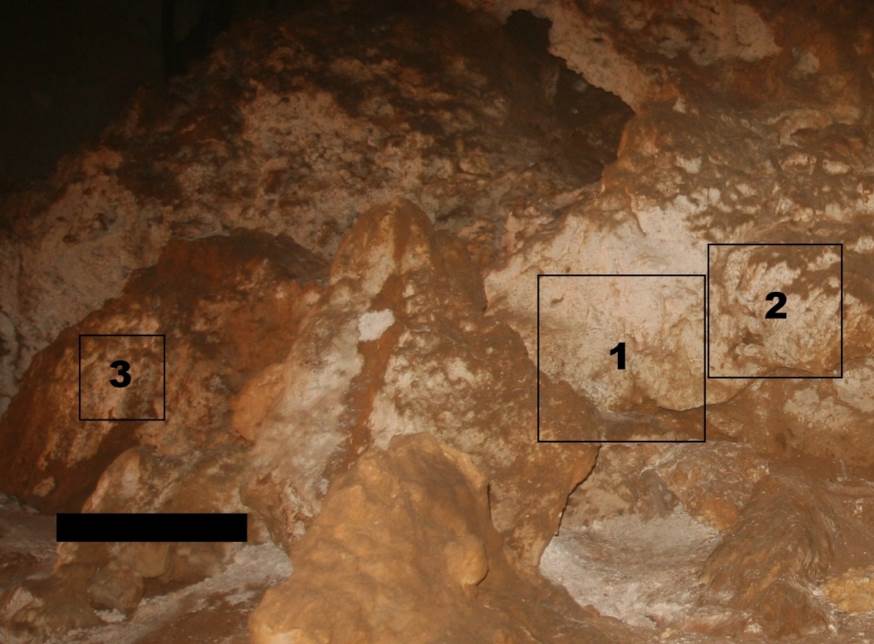


CRP North is distinct from the other CRP regions, being composed of isolated boulders rather than a continuous rock face. Each boulder has distinct characteristics and most bear claw marks. Mark preservation varies presumably due to the differences in surface morphologies. This meant that marks were generally confined to smaller areas allowing preservation of 25 scratch sets.

| **Supplementary Figure 25 | Claw-marked surface, CRP North 1.** (**A**) Original photograph. (**B**) Identified scratch sets. Scale bar = 10 cm. | **Supplementary Figure 26 | Claw-marked surface, CRP North 2.** (**A**) Original photograph. (**B**) Identified scratch sets. Scale bar = 10 cm. |
| --- | --- |
| 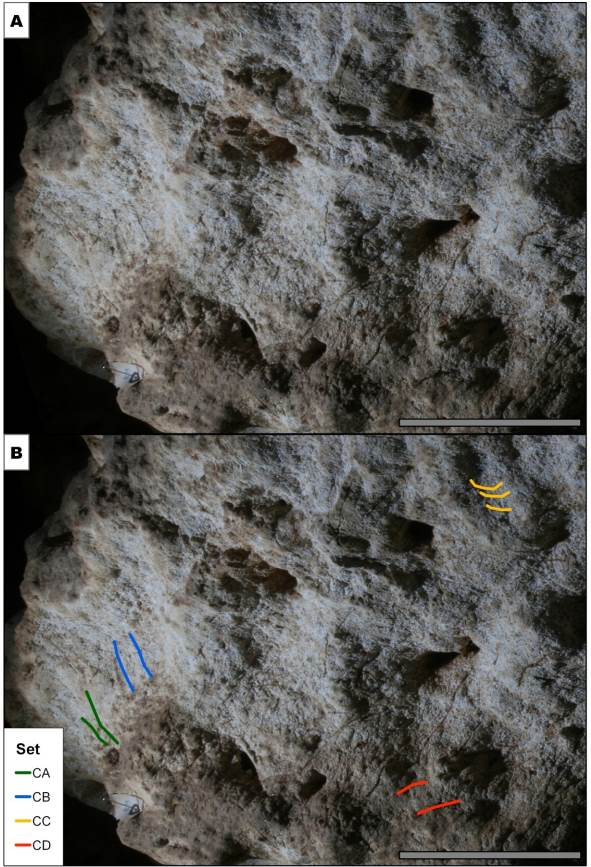 | 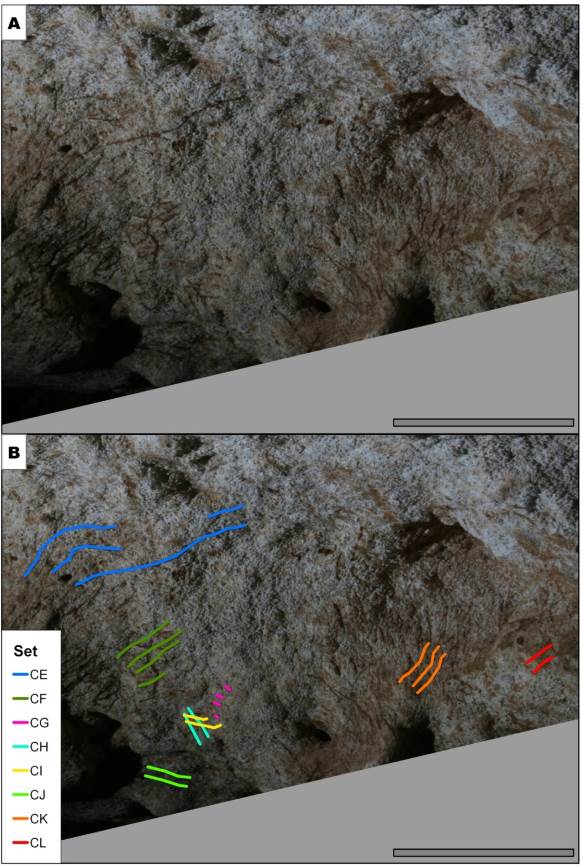 |

| **Supplementary Figure 27 | Claw-marked surface, CRP North 3.** (**A**) Original photograph. (**B**) Identified scratch sets. Scale bar = 10 cm. | **Supplementary Figure 28 | Claw-marked surface, CRP North 4.** (**A**) Original photograph. (**B**) Identified scratch sets. Scale bar = 10 cm. |
| --- | --- |
| 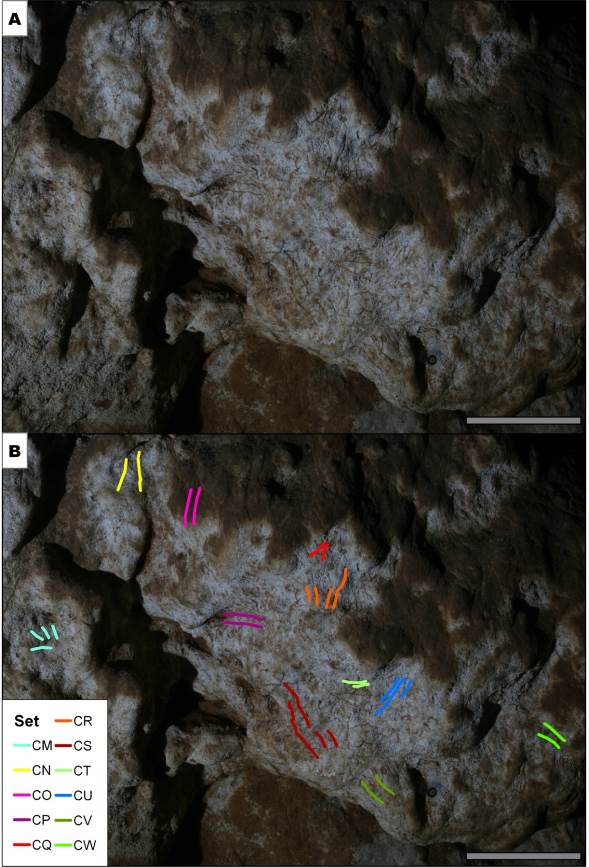 | **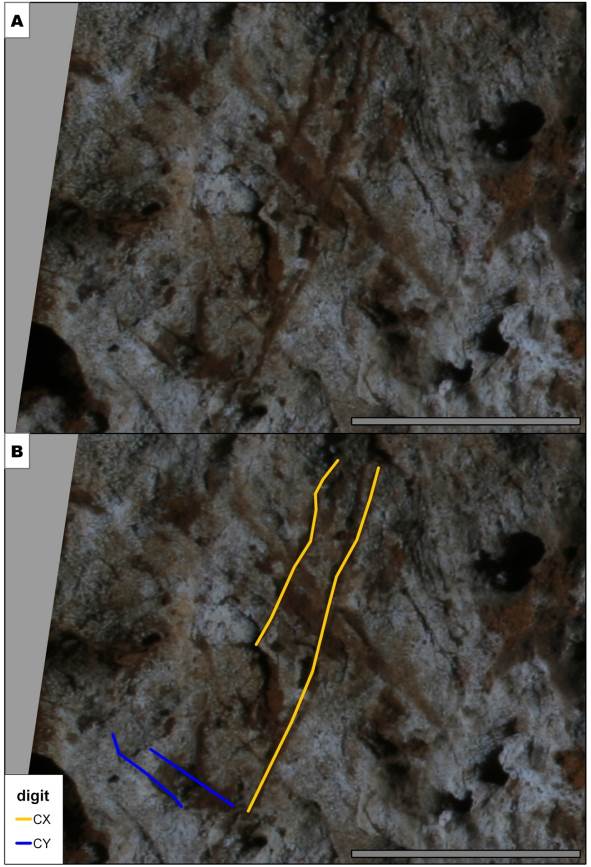** |

Claw marks on the top face of the CRP (CRP Top) were considered as a distinct group primarily due to their spatial separation from the other region. The surface is continuous but highly angular. Two distinct scratch sets were identified on this hard, sediment-encrusted surface, although other marks were sporadically distributed across this region.

**Supplementary Figure 29 | Claw-marked surface, CRP Top.** (**A**) Original photograph. (**B**) Identified scratch sets. Scale bar = 10 cm.

1.
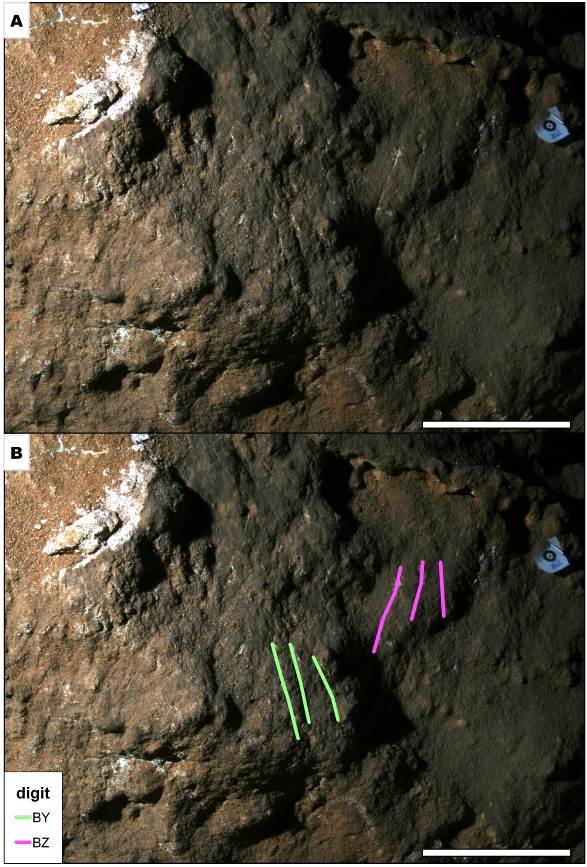


*Inter-digital Spacing Distribution*

The distribution of IDS values across TEC was non-normal with a positive skew (Fig. 3). This was supported by a Shapiro–Wilk test that refuted the null hypothesis of a normal distribution (*p* < 0.000). The scratch mark length histogram also appeared positively skewed, which was again supported by a Shapiro–Wilk test where a normal distribution was rejected (*p* < 0.000). The Kruskal–Wallis test revealed both IDS and set length to be significantly different between regions in TEC (both *p* < 0.000). Mann–Whitney post-hoc results reveal significant differences between most regions for IDS, and to a lesser extent set length (Supplementary Fig. 11). However, this may be partly due to low sample sizes. Considering orientation of scratch mark sets, the Kruskal–Wallis test again indicated significant differences between regions (*p =* 0.002).

**Supplementary Table 11 | Mann–Whitney pairwise comparisons across the Tight Entrance Cave claw-marked regions.**

| **Inter-digital Spacing**  **Set Length** | **Cave Wall South** | **Boulder** | **CRP West** | **CRP South** | **CRP North** | **CRP Top** |
| --- | --- | --- | --- | --- | --- | --- |
| CWS | – | 0.580 | 0.064 | 0.021 | 0.013 | 0.006 |
| Boulder | 0.315 | – | 0.054 | < 0.000 | < 0.000 | 0.007 |
| CRP west | 0.300 | 0.677 | – | < 0.000 | < 0.000 | 0.245 |
| CRP south | 0.885 | < 0.000 | 0.008 | – | 0.809 | < 0.000 |
| CRP north | 0.736 | < 0.000 | 0.005 | 0.508 | – | < 0.000 |
| CRP top | 0.773 | 0.978 | 1.000 | 0.054 | 0.084 | – |

Similarly, much of the variation in claw marking noted between regions within TEC may be attributable to variation in hardness, inclination and morphology of the limestone surfaces on which marks were made. Hardness, in particular, appears to be a major factor affecting markings, with markings on soft or sediment coated limestone often straighter and simpler, possibly due to soft surfaces offering less resistance to claws than harder limestone. Complex surface morphology, however, is likely to force the animals to move their limbs and/or digits to ensure that they retain contact with the substrate. Evidence for surface morphology being the primary cause of the variation seen is exhibited in the regional variation analysis, which showed CRP North and South to be similar to one another but distinguishable from all other regions. These regions are similar in their surface morphology, which is often highly irregular, supporting climbing being the primary cause of this variation.

**Attribution Process**

In the comparative study, normal distribution of IDS data was considered indicative of a monospecific dataset. The distribution of IDS values across TEC was non-normal with a positive skew, which was supported by a Shapiro–Wilk test that refuted the null hypothesis of a normal distribution (*p* < 0.000). Scratch-mark length is also positively skewed, which was supported by a Shapiro–Wilk test where a normal distribution was rejected (*p* < 0.000). The Kruskal–Wallis test revealed IDS and set length to be significantly different between regions in TEC (both *p* < 0.000).

**Taphonomic Data**

The following tables contain data that underpin the taphonomic analysis of the Tight Entrance Cave bone deposit, specifically surface marks evidently made by biological agents.

**Supplementary Table 12 | Criteria used for analysis of Tight Entrance Cave bone modification by biological agents.** Dasyurid category focuses upon criteria observed for *Sarcophilus harrisii* but includes a few smaller marks possibly made by *Dasyurus geoffroii*, but tooth marks left on bone by living individuals of this species have never been characterised.

| **Agent** | **Observation** | **Supporting Knowledge** | **Reference** |
| --- | --- | --- | --- |
| Dasyurid | Markings focused on trabecular bone | *Sarcophilus* preference for trabecular bone | Sobbe 1990 |
|  | Markings focused on epiphyses, limb bone ends | *Sarcophilus* preference for chewing ends off limb bones | Marshall & Cosgrove 1990 |
|  | Marks surrounding exposed edges of bone | *Sarcophilus* preference for chewing ends off limb bones | Marshall & Cosgrove 1990 |
|  | Conical markings found on opposing sides of bone | Dentary and maxilla in jaws leaving marks on opposing sides of bone; tri-cuspid molars | Sobbe 1990 |
|  | Crushing of puncture edges | Large forces involved in bite crush edges | Sobbe 1990 |
|  | Puncturing | To distinguish from acid etching where no other evidence present | Sobbe 1990 |
| *Thylacoleo carnifex* | Longitudinal marks | Blade-like premolar producing longitudinal marks | Runnegar 1983 |
|  | Longitudinal marks on opposed edges | Dentary and maxilla in jaws leaving marks on opposing sides of bone; sectorial premolar | Horton & Wright 1981 |
|  | Flake removed | Angled force chipping flake of cortical bone off | Sobbe 1990 |
| Rodents | Parallel Grooves | Paired incisors producing parallel grooves | Haglund et al.1988 |
|  | Wedges removed | Rodent gnawing removing wedges of bone | Haglund et al.1988 |
| Termites | Tunneling | Tunneling through cortical bone to access favored trabecular bone | Huchet et al. 2011 |
|  | Trabecular bone removed | Trabecular bone favored by termites | Huchet et al. 2011 |
| Depositional | Isolated punctures | To distinguish from *Sarcophilus* as predators unlikely to leave single markings | Andrews 1990 |
|  | Perfectly circular | Drip-water erosion leaving circular marks | Andrews 1990 |
|  | Root still present | Roots penetrating through bone seeking water | Sobbe 1990 |
|  | Meandering root markings | Organic acids etching bone with root patterns distinguishable | Sobbe 1990 |

**Supplementary Table 13 | Reliability index indicating degree of confidence with which markings on Tight Entrance Cave bones have been attributed to biological agent.**

| **Index** | **Reliability** |
| --- | --- |
| 1 | Poor – Markings noted but no certainty as to taphonomic agent |
| 2 | Adequate – Lowest level at which taphonomic agent could be indicated |
| 3 | Good – Markings well preserved and distinctive to perpetrator |
| 4 | Excellent – Highest quality preservation of distinctive markings |

**Supplementary Table 14 | Number and percentages of specimens in the Tight Entrance Cave bone assemblage noted to have been modified by biological agents grouped according to their Reliability Index.**

| **Agent** | **Reliability Index** | | | | | | | |
| --- | --- | --- | --- | --- | --- | --- | --- | --- |
|  | **1** | | **2** | | **3** | | **4** | |
|  | **No.** | **% total** | **No.** | **% total** | **No.** | **% total** | **No.** | **% total** |
| Dasyurid | 53 | 33.8 | 59 | 37.6 | 38 | 24.2 | 7 | 4.5 |
| *Thylacoleo* | 4 | 66.7 | 2 | 33.3 | 0 | 0.0 | 0 | 0 |
| Rodent | 7 | 30.4 | 12 | 52.2 | 4 | 17.4 | 0 | 0 |
| Termite | 62 | 36.9 | 75 | 44.6 | 29 | 17.3 | 2 | 1.19 |
| Depositional | 21 | 60.0 | 10 | 28.6 | 3 | 8.6 | 1 | 2.86 |

**Supplementary Table 15 | Biologically-modified specimens (with Reliability Index 2–4) organised by unit.** Relative proportion is the total number of attributed to a particular agent divided by the total number of specimens examined (10,921) and expressed as a percentage.

| **Unit**  **Agent** | **B** | **D** | **E** | **E*** | **F1** | **F2** | **G** | **H** | **J** | **Total** | **Relative Proportion (%)** |
| --- | --- | --- | --- | --- | --- | --- | --- | --- | --- | --- | --- |
| Dasyurid | 17 | 44 | 1 | 9 | 5 | 5 | 3 | 8 | 11 | 103 | 0.97 |
| *Thylacoleo* | 1 | 1 | 0 | 0 | 0 | 0 | 0 | 0 | 0 | 2 | 0.02 |
| Rodent | 4 | 9 | 0 | 0 | 0 | 0 | 0 | 0 | 3 | 16 | 0.15 |
| Termite | 9 | 78 | 0 | 4 | 2 | 3 | 0 | 2 | 8 | 106 | 1.00 |
| Depositional | 3 | 3 |  | 1 | 1 | 1 | 1 | 0 | 4 | 14 | 0.13 |
| Total | 34 | 135 | 1 | 14 | 8 | 9 | 4 | 10 | 26 | 241 | 2.27 |

**Supplementary Table 16 | List of specimens bearing tooth marks of Dasyurids with Reliability Index 3–4.**

| **WAM No.** | **Taxonomic Identification** | **Element(s)** | **Unit** | **Reliability Index** |
| --- | --- | --- | --- | --- |
| 09.3.75 | Macropodidae sp. indet. | right femur proximal end | B | 3 |
| 09.3.86 | Macropodidae sp. indet. | left femur proximal end | B | 3 |
| 09.3.85 | Macropodidae sp. indet. | right tibia proximal end | B | 3 |
| 09.3.87 | Macropodidae sp. indet. | right tibia proximal end | B | 3 |
| 09.3.92 | Macropodinae sp. indet. | right femur | B | 3 |
| 09.3.80 | Macropodidae sp. indet. | right tibia proximal end | B | 3 |
| 97.5.49 | *Procoptodon browneorum* | left dentary | B | 3 |
| 97.5.97 | *Sarcophilus harrisii* | left dentary | B | 3 |
| 97.5.98 | *Sarcophilus harrisii* | right dentary | B | 4 |
| 09.3.188 | Macropodinae sp. indet. | tibia shaft | D | 3 |
| 09.3.131 | Macropodidae sp. indet. | tibia shaft | D | 3 |
| 09.3.129 | Macropodidae sp. indet. | right tibia proximal end | D | 3 |
| 09.3.130 | Macropodidae sp. indet. | left tibia proximal end | D | 3 |
| 09.3.189 | Macropodinae sp. indet. | right femur | D | 4 |
| 09.3.119 | Macropodidae sp. indet. | left humerus proximal end | D | 3 |
| 09.3.199 | Macropodinae sp. indet. | left femur proximal end | D | 3 |
| 09.3.154 | Macropodidae sp. indet. | left femur proximal end | D | 3 |
| 09.3.156 | Macropodidae sp. indet. | right tibia proximal end juvenile | D | 3 |
| 09.3.155 | Macropodidae sp. indet. | limb bone fragment | D | 3 |
| 09.3.173 | Macropodinae sp. indet. | left femur distal end | D | 3 |
| 09.3.125 | Macropodidae sp. indet. | right tibia, proximal end | D | 3 |
| 09.3.205 | Mammalia sp. indet. | right femur distal fragment | D | 3 |
| 09.3.149 | Macropodidae sp. indet. | thoracic vertebra | D | 4 |
| 09.3.174 | Macropodinae sp. indet. | left metatarsal IV | D | 4 |
| 97.5.237 | *Procoptodon browneorum* | left dentary | D | 4 |
| 99.11.597 | Simosthenurini | left I3; left i1; right i1 | E | 3 |
| 09.3.225 | Macropodidae sp. indet. | left tibia proximal end | E* | 3 |
| 09.3.232 | Macropodinae sp. indet. | left ischium | E* | 3 |
| 09.3.229 | Macropodinae sp. indet. | right tibia shaft | E* | 3 |
| 08.8.470 | *Zygomaturus trilobus* or Vombatidae | tibia fragment proximal portion | E* | 3 |
| 08.1.99 | *Macropus fuliginosus* | left femur distal epiphysis | F1 | 3 |
| 08.1.443 | *Macropus fuliginosus* | distal half of right tibia | F2 | 4 |
| 08.1.444 | Simosthenurini sp. indet. | partial innominate | F2 | 3 |
| 08.1.55 | *Procoptodon browneorum* | medial podial phalanx IV | F2 | 3 |
| 99.11.471 | *Simosthenurus occidentalis* | left and right dentaries | F2 | 4 |
| 04.2.14 | *Procoptodon browneorum* | left dentary | G | 3 |
| 04.2.69 | *Procoptodon browneorum* | right dentary | G | 3 |
| 07.6.147 | *Procoptodon browneorum* | partial skeleton | G | 3 |
| 09.3.239 | Macropodidae sp. indet. | left femur juvenile | H | 3 |
| 09.3.240 | Macropodidae sp. indet. | left metatarsal IV | H | 3 |
| 08.1.424 | *Procoptodon browneorum* | distal manual phalanx | H | 3 |
| 08.8.520 | Marsupialia sp. indet. | ischium bone fragment | H | 3 |
| 09.3.244 | Macropodinae sp. indet. | right tibia shaft | J | 3 |
| 07.6.8 | *Trichosurus vulpecula* | left dentary | J | 3 |
| 08.8.513 | *Macropus fuliginosus* | femur distal articulation | J | 3 |

***Supplementary Table 17 | List of all specimens bearing potential tooth marks of Thylacoleo carnifex***

| **WAM No.** | **Taxonomic Identification** | **Element(s)** | **Unit** | **Reliability Index** |
| --- | --- | --- | --- | --- |
| 09.3.96 | Sthenurinae sp. indet | right tibia shaft | B | 2 |
| 09.3.124 | Macropodidae sp. indet. | right tibia shaft | D | 2 |
| 99.11.23 | Macropus fuliginosus | left dentary | D | 1 |
| Bulk collection | Macropodidae sp. indet. | left juvenile tibia distal end | B | 1 |
| Bulk collection | Macropodidae sp. Indet. | left tibia shaft | D | 1 |
| Bulk collection | Mammalia sp. Indet. | fragment | D | 1 |

**Supplementary** **Figure 30 | Examples of bones from Tight Entrance Cave modified by biological agents.** (**A**) WAM 08.1.443, *Sarcophilus* tooth marks on opposing sides of bone. (**B**) WAM 09.3.125, termite and *Sarcophilus* tooth marks on opposing side of bone; epiphysis removed and trabecular bone, tunnelling. (**C**) WAM 09.3.105, rodent tooth marks denoted by parallel grooves, wedge of bone removed. (**D**) Left to right: WAM 09.3.179, 09.3.186, 09.3.140, termite modified; tunnelling, trabecular bone removed. (**E**) Left to right: WAM 09.3.124, 09.3.96, ?*Thylacoleo carnifex* tooth marks; longitudinal marks, marks on opposing side of bone, flake of cortical bone removed. Scale bars = 20 mm.

**References**

Andrews, P. *Owls, caves and fossils: predation, preservation and accumulation of small mammal bones in caves, with an analysis of the Pleistocene cave faunas from Westbury-sub-Mendip, Somerset, UK* (University of Chicago Press, 1990).

Bednarik, R. On natural cave markings. *Helictite* **29,** 27–41 (1991).

Bednarik, R. The speleothem medium of ﬁnger ﬂutings and its isotopic geochemistry. *The Artefact* **22**, 49-64 (1999).

Borsato, A., Frisia, S., Jones, B., & van der Borg, K. Calcite Moonmilk: Crystal Morphology and Environment of Formation in Caves in the Italian Alps. *Journal of Sedimentary Research* **70,** 1171-1182 (2000).

Finch, M. E. & Freedman, L. Functional morphology of the limbs of *Thylacoleo carnifex* Owen (Thylacoleonidae: Marsupialia). *Aust. J. Zool.* **36,** 251–272 (1988).

Gobetz, K. E. Possible burrows of mylagaulids (Rodentia: Aplodontoidea: Mylagaulidae) from the late Miocene (Barstovian) Pawnee Creek Formation of northeastern Colorado. *Palaeogeog. Palaeoclim. Palaeoecol.* **237,** 119–136 (2006).

Haglund, W. D., Reay, D. T. & Swindler D. Tooth mark artifacts and survival of bones in animal scavenged human skeletons. *J. Foren. Sci.* **33,** 985–997 (1988).

Horton, D. R. & Wright, R. V. S. Cuts on Lancefield bones: carnivorous *Thylacoleo*, not humans, the cause. *Archaeol. Oceania* **16,** 73–80 (1981).

Huchet, J. B., Deverly, D., Gutierrez, B. & Chauchat, C. Taphonomic evidence of a human skeleton gnawed by termites in a Moche-civilisation grave at Huaca de la Luna, Peru. *Int. J. Osteoarchaeol.* **21,** 92–102 (2011).

Lundelius, E. L. Marsupial carnivore dens in Australian caves. *Studies Speleo.* **1,** 174–181 (1966).

Marshall, B. & Cosgrove, R. Tasmanian Devil (*Sarcophilus harrisii*) scat-bone: signature criteria and archaeological implications. *Archaeol. Oceania* **25,** 102–113 (1990).

Murray, P. F. in *Wombats* (eds Wells R. T. & Pridmore, P. A.) 1–33 (Surrey Beatty & Sons, 1998).

Owen, D. & Pemberton, D. *Tasmanian Devil: A Unique and Threatened Animal* (Allen & Unwin, 2005).

Paddle, R. N. *The Last Tasmanian Tiger: The History and Extinction of the Thylacine*. (Cambridge Univ. Press, 2000).

Prideaux, G. J. *et al.* Timing and dynamics of Late Pleistocene mammal extinctions in southwestern Australia. *Proc. Natl Acad. Sci. USA* **107,** 22157–22162 (2010).

Runnegar, B. A. *Diprotodon* ulna chewed by the marsupial lion, *Thylacoleo carnifex*. *Alcheringa* **7,** 281–295 (1983).

Sobbe, I. H. 1990. Devils on the Darling Downs - the Tooth mark Record. Memoirs of the Queensland Museum 27(2):299-322.Triggs, B. *Tracks, scats and other traces: a field guide to Australian mammals* (Oxford University Press, 2004).

van Dyck, S. & Strahan, R. *The Mammals of Australia, 3^rd^ ed.* (Reed New Holland, 2008).

Wells, R. T., Horton, D. R. & Rogers, P. in *Carnivorous Marsupials* (ed. Archer, M.) 201–228 (Royal Zoological Society of New South Wales, 1982).

Wells, R. T. & Nicol, B. On the manus and pes of *Thylacoleo carnifex* Owen (Marsupialia). *Trans. R. Soc. S. Aust.* **101,** 139–146 (1977).
